# *Piriformospora indica* Primes Onion Response against Stemphylium Leaf Blight Disease

**DOI:** 10.3390/pathogens10091085

**Published:** 2021-08-26

**Authors:** Praveen Roylawar, Kiran Khandagale, Pragati Randive, Bharat Shinde, Chandrashekhar Murumkar, Avinash Ade, Major Singh, Suresh Gawande, Massimiliano Morelli

**Affiliations:** 1 ICAR-Directorate of Onion and Garlic Research (DOGR), Rajgurunagar, Pune 410505, India; roylawar@sangamnercollege.edu.in (P.R.); randivepragati@gmail.com (P.R.); major.singh@icar.gov.in (M.S.); 2 Tuljaram Chaturchand College of Arts, Science and Commerce, Baramati, Pune 413102, India; drcvmurumkar@gmail.com; 3 Department of Botany, Sangamner Nagarpalika Arts, D. J. Malpani Commerce, B. N. Sarda Science College, Sangamner, Ahamadnagar 422605, India; 4 Department of Botany, Savitribai Phule Pune University, Pune 411007, India; kirankhandagale253@gmail.com (K.K.); avinashade@unipune.ac.in (A.A.); 5 Vidya Pratishthan’s Arts, Science & Commerce College, Baramati, Pune 413133, India; principal.vpascc@vidyapratishthan.com; 6 CNR-IPSP Istituto per la Protezione Sostenibile delle Piante, Sede Secondaria di Bari, 70124 Bari, Italy; massimiliano.morelli@ipsp.cnr.it

**Keywords:** biocontrol, Induced systemic resistance, onion, *Piriformospora indica*, Stemphylium leaf blight disease, *Stemphylium vesicarium*

## Abstract

The root-endophytic fungus *Piriformospora indica* (=*Serendipita indica*) has been revealed for its growth-promoting effects and its capacity to induce resistance in a broad spectrum of host plants. However, the bioefficacy of this fungus had not yet been tested against any pathogen affecting onion (*Allium cepa*). In this study, the biocontrol potency of *P. indica* against onion leaf blight, an impacting disease caused by the necrotrophic fungal pathogen *Stemphylium vesicarium*, was evaluated. First, it was proved that colonisation of onion roots by *P. indica* was beneficial for plant growth, as it increased leaf development and root biomass. Most relevantly, *P. indica* was also effective in reducing Stemphylium leaf blight (SLB) severity, as assessed under greenhouse conditions and confirmed in field trials in two consecutive years. These investigations could also provide some insight into the biochemical and molecular changes that treatment with *P. indica* induces in the main pathways associated with host defence response. It was possible to highlight the protective effect of *P. indica* colonisation against peroxidative damage, and its role in signalling oxidative stress, by assessing changes in malondialdehyde and H_2_O_2_ content. It was also showed that treatment with *P. indica* contributes to modulate the enzymatic activity of superoxide dismutase, catalase, phenylalanine ammonia-lyase and peroxidase, in the course of infection. qPCR-based expression analysis of defence-related genes *AcLOX1*, *AcLOX2*, *AcPAL1*, *AcGST*, *AcCHI*, *AcWRKY1,* and *AcWRKY70* provided further indications on *P. indica* ability to induce onion systemic response. Based on the evidence gathered, this study aims to propose *P. indica* application as a sustainable tool for improving SLB control, which might not only enhance onion growth performance but also activate defence signalling mechanisms more effectively, involving different pathways.

## 1. Introduction

**Figure 1:**
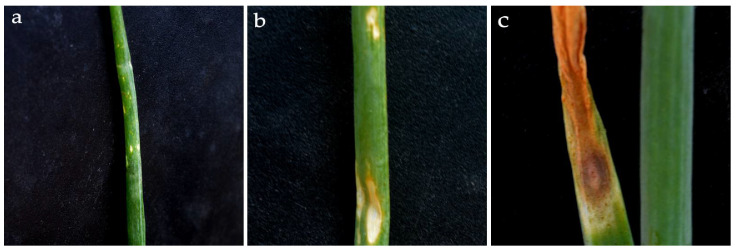
Development of Stemphylium leaf blight disease on onion plants following infection by *S. vesicarium*. (**a**) Initial symptoms consisting of white/yellowish spots; (**b**) more evident spots with purple/brown border and yellowing of the leaf tips; (**c**) severe symptoms with large necrotic lesions and foliar desiccation.

*Stemphylium vesicarium* (Wallr.) E. Simmons [1] is the most common destructive foliar fungal disease of onion (*Allium cepa*) worldwide [2,3,4,5]. Besides onion, this pathogen also infects a wide range of other economically important crops such as garlic (*Allium sativum*) [6], chilli pepper (*Capsicum annuum*) [7], leek (*Allium ampeloprasum*) [6], asparagus (*Asparagus officinalis*) [8], pear (*Pyrus communis*) [9,10], and mango (*Mangifera indica*) [11]. In onion, *S. vesicarium* causes Stemphylium leaf blight disease (SLB) [12]. Typically, SLB occurrence becomes evident as the plant comes to maturity and the leaf senescence process begins. Symptoms initially limited to characteristic yellowish, water-soaked spots progress rapidly as the pathogen commences to release its spores. The leaves then begin to develop lesions that become progressively more extensive, until they coalesce and result in large necroses that can lead to complete leaf desiccation and plant death [13] (Figure 1).

Although in onion, as in garlic, the infection usually remains confined to the leaves, and rarely colonises the bulb, SLB is leading to significant yield losses in several countries [14,15,16,17,18]. One of the most severely affected areas is India, where losses of up to 100% of onion production have been reported, due to its widespread favoured by suitable environmental conditions [19].

Based on current knowledge, there are no SLB-resistant onion varieties available on the market, and although with different degrees of susceptibility, all tested cultivars succumb to *S. vesicarium* infection, as demonstrated in studies conducted in distinct geographic areas [20,21]. Methods for cultural management of SLB have been proposed, mainly based on reduction of pathogen inoculum on vegetation debris by tillage or in the soil by crop rotation and controlled irrigation practices to allow rapid leaf drying [13]. However, all those strategies appear to be useful in integrated management of the disease, but have proven to be a palliative treatment towards the pathogen. Most recently Leach et al. [22] have reported that the co-occurrence of onion thrips (*Thrips tabaci*) may exacerbate the damage induced by *S. vesicarium* and contribute to propagate its conidia. Therefore, they have proposed thrips control as an effective strategy to limit SLB incidence. However, this study is still at a preliminary stage and far from being deployed at a commercial scale.

Due to a lack of knowledge on reliable traits of host resistance and the non-availability of robust alternative strategies for SLB management and containment, a widespread, and often unrestrained use of chemical fungicides is the method most often recurred for managing SLB disease at different latitudes.

As shown in other pathosystems, heavy use of synthetic fungicides is not only increasing the cost of production but also aiding and abetting human and environmental hazards. Additionally, the extensive use of synthetic fungicides results in increasing pathogen mutations and resistance to various active principles [23]. This is also the case for SLB whose emergence in New York state has been recently associated with the rise of resistant *S. vesicarium* populations, due to the intensive use of fungicides targeting foliar disease in onion fields [24]. Raising awareness of these adverse effects, have fostered an unprecedented interest in the use of biocontrol agents, as a proven alternative strategy for chemical control [25]. Besides antagonism, biocontrol agents are known for inducing resistance in plants and thereby leading to sustainable, healthy, and enhanced prospects to manage disease occurrence. Among these defence mechanisms, induced systemic resistance (ISR) develops as a result of beneficial interaction between host and beneficial microbes, often inhabiting the rhizosphere [26]. This finely regulated process results in improved plant growth performances and resistance against a wide range of biotic and abiotic stresses [27,28].

The use of biocontrol agents has been sparsely investigated against *S. vesicarium*-associated diseases, with few beneficial microbes including *Bacillus subtilis*, *Pseudomonas fluorescences,* and *Trichoderma* spp. so far being reported to show promising results in disease management [29,30,31]. However, these studies were lacking in-depth details of molecular-level interactions of these biocontrol agents with host and their target pathogens.

*Piriformospora indica*(=*Serendipita indica*) is a root-endophytic basidiomycete member of the order Sebacinales [32], which was originally isolated from a spore of the arbuscular mycorrhizal fungus (AMF) *Glomus mosseae*, in the remote desert of Thar, northwestern India [33]. Differently from AMF, with which it shares some biological features, *P. indica* can be easily grown in axenic culture [34] and it has shown remarkable effects on growth promotion and tolerance to environmental stresses in a wide array of mono- and dicotyledonous host plants [35,36,37].

In recent years it has also been reported that *P. indica* is effective in boosting plant resistance and has been proposed as a promising biocontrol agent against a wide range of foliar and root pathogens [37,38], also proved able to protect roots from herbivores [39]. It could be hypothesised that, in the case of root pathogens, *P. indica* may exert a direct antagonistic effect. However, this inhibitory effect has been scarcely observed and only reported, to a limited extent, against *Fusarium oxysporum*, in Fusarium wilt-diseased lentil (*Lens culinaris*) [40]. Increasing evidence collected in plants colonised by *P. indica*, on its effects against leaf pathogens, led to assume that the fungus is able to induce systemic resistance, as it was demonstrated in barley [41], wheat [42], maize [43], tomato [44] and *Arabidopsis thaliana* [45]. In particular, the discovery that *P. indica* successfully colonises and interacts with *A. thaliana*, has been providing a valid model to hypothesise which genes may play a role in its beneficial interaction with plants [46]. The *ability* of *P. indica* to trigger plant response against fungal pathogens, which is preferable to an ISR-like mechanism of action, has been related to jasmonic acid (JA)-dependent signalling [45], to the synthesis of antioxidant agents [36,47] and the regulation of gene expression in defence-related pathways [44,48,49]. It has been shown that endofungal bacteria like *Rhizobium radiobacter* F4 are always present inside *P. indica*, at least in low numbers, producing N-acyl homoserine-lactone (AHLs) and showing plant beneficial effects without the fungus [50,51]. Although these mechanisms have not yet been fully elucidated, evidence gathered on the efficacy of *P. indica* and its high rates of successful colonisation have paved the way for its wide use as a growth promoter and biocontrol agent [52]. Up to now, however, the efficacy of *P. indica* against SLB disease in onion had not yet been evaluated.

Through this study, we attempted to use *P. indica* as a biocontrol agent against *S. vesicarium* and characterised the effects of its colonisation on host responses, in a highly susceptible onion cultivar. We also compared the field performance of *P. indica* treatment with chemical fungicides commonly used against SLB. Moreover, the impact of *P. indica* treatment on plant fitness, its biochemical effect on key enzymatic content, and changes in expression of defence marker genes were also investigated in this study.

To our knowledge, this is the first attempt to explore the defence response triggered by *P. indica* against onion fungal pathogens and aims to provide a significant contribution to the biological control of a detrimental disease highly impacting onion-producing areas.

## 2. Results

### 2.1. Colonisation of Onion Roots by P. indica

The ability of *P. indica* to establish successful colonisation of onion roots was preliminarily assessed by microscopy observations at 45 days post-treatment. Pear-shaped chlamydospores were observed in the onion root cortex, confirming that the hyphae of the fungus had successfully penetrated the epidermal layers. In control plants, no colonisation was observed. Evidence provided by microscopy visualisation was further confirmed by molecular detection through PCR. An amplicon of the expected size (84 bp), corresponding to the *Pitef1* gene of *P. indica*, was successfully amplified from cDNA of plants exposed to the fungus, and not from controls.

### 2.2. Effects of P. indica Colonisation on Onion Growth Parameters

The physiological effect of *P. indica* treatment on relevant onion growth parameters was evaluated at 60 days post-treatment under greenhouse conditions (Figure S1, Table S1). When compared with untreated controls, plants exposed to *P. indica* showed a significant increase in growth performance, as measured by leaf length (+86.7%, *p* < 0.05). The presence of the fungus also resulted in a significant increment in dry (+84.0%, *p* < 0.01) and fresh (+83.8%, *p* < 0.01) root weight. A less pronounced and nonsignificant increase was measured in root length (+6.1%, *p* > 0.05). Treatment with *P. indica* did not induce any observable phytotoxic effects or phytopathological damage to leaf or bulb parts.

### 2.3. Efficacy of P. indica against Stemphylium Leaf Blight Disease

#### 2.3.1. Greenhouse Trial

**Figure 2:**
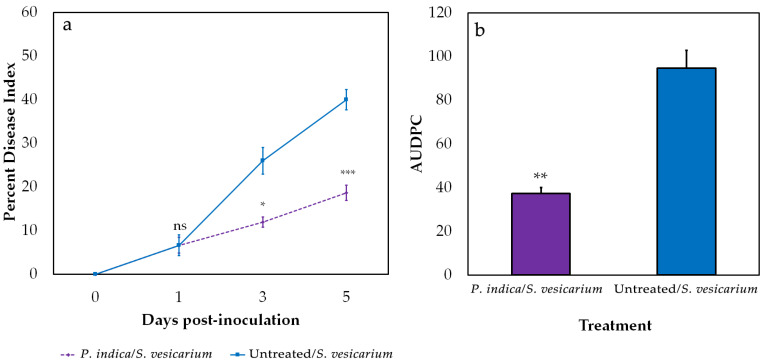
Effects of *P. indica* treatment on onion Stemphylium leaf blight disease, in greenhouse conditions. Percent disease index (PDI) (**a**) was scored at 1, 3, 5 days post-inoculation with *S. vesicarium* and compared between *P. indica*-treated and untreated plants. The area under disease progress curve (AUDPC) (**b**) was calculated based on the PDI plot. PDI and AUDPC values are expressed as the average of three biological replicates, each consisting of ten plants pooled together. Statistically significant differences were assessed by Student’s *t*-test at a 5% significance level. *p*-value significance codes: ‘***’ < 0.001, ‘**’ < 0.01, ‘*’ < 0.05, ‘ns’ not significant. Error bars indicate the standard error of the mean.

The efficacy of *P. indica* in attenuating the development of SLB symptoms on onion leaves was evaluated under greenhouse conditions at 1, 3, and 5 days post-inoculation (dpi) with *S. vesicarium*. The effects on disease progress were scored in terms of Percent Disease Index (PDI), based on visual observations. Although no differences between treated and untreated plants were observed at 1 dpi, successive observations showed a significant decrease in *S. vesicarium*-induced symptoms in plants colonised by *P. indica*, clearly evident at 3 dpi (−53.8%) and 5 dpi (−53.3%). (Figure 2a, Table S2). The overall effect on SLB symptom reduction was also highlighted by the area under disease progress curve (AUDPC) values, significantly lower in *P. indica*-treated plants (−67.1%, *p* < 0.05) (Figure 2b, Table S3). Taken together, these results showed that *P. indica* is able to moderate the development of symptoms caused by *S. vesicarium* in onion plants grown under greenhouse conditions.

#### 2.3.2. Field Trial

**Figure 3:**
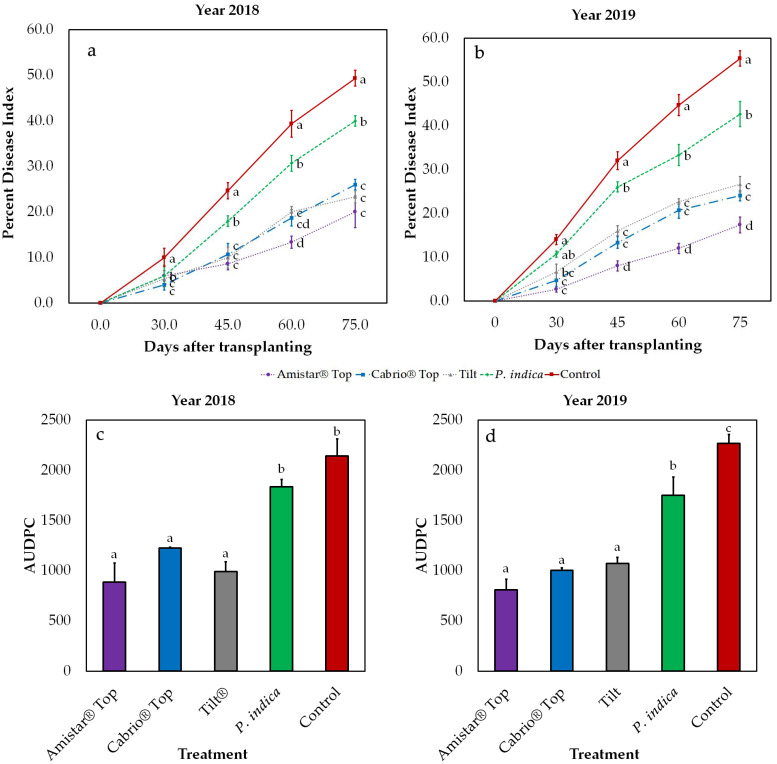
Effects of *P. indica* treatment on onion Stemphylium leaf blight disease, in field conditions. Percent disease index (PDI) was scored in two independent trials in 2018 (**a**) and 2019 (**b**) at 30, 45, 60, 75 days after field transplanting and compared among plants treated with *P. indica*, treated with three different fungicides (Amistar^®^ Top, Cabrio^®^ Top and Tilt^®^) or untreated. The area under disease progress curve (AUDPC) was calculated based on PDI plots for 2018 (**c**) and 2019 (**d**). Values for each year are expressed as the average of three biological replicates, each consisting of ten plants pooled together. Statistically significant differences were assessed by one-way analysis of variance (ANOVA) followed by a post hoc Duncan’s multiple range test. Different letters indicate statistically significant differences (*p*-value < 0.05). Error bars indicate the standard error of the mean.

We conducted two field observations, in the years 2018 and 2019, to measure the effect of *P. indica* treatment on plants naturally exposed to *S. vesicarium* infection. The development of SLB symptoms was measured in terms of PDI, every 15 days after transplanting in the open field. In both trials *P. indica* was effective in mitigating the effects of SLB, presenting a statistically significant reduction in disease severity compared to the untreated control, which ranged from 20.90 to 31.90%, averaging between the two experiments and the different time points (Figure 3a,b, Table S4). We also included in the experiment a comparison with the three fungicides most commonly used for SLB disease control in the area (Cabrio^®^ Top, Tilt^®^ and Amistar^®^ Top). Amistar^®^ Top proved to be the most effective in reducing disease severity, in both repeats of the experiment.

AUDPC values were lower in plants treated with *P. indica* than in the controls, although this reduction was more pronounced in 2019 (−22.9%) than in 2018 (−14.5%) (Figure 3c,d, Table S5). Based on AUDPC values, the performance of the three fungicides did not differ significantly from each other. AUDPC analysis also allowed us to assess the relative efficacy of *P. indica* compared to the three commercial products. Based on this estimation, our biological treatment provided an efficacy ranging from 46% of the effect achieved with Amistar^®^ Top (2019) to 67% of that obtained with Cabrio^®^ Top (2018).

A linear model (lm) analysis allowed us to rule out the existence of a significant random effect due to the trial season. We established two models containing ‘Year’ and ‘Biological Replicate’ as random factors while ‘Treatment’ and ‘PDI’ or ‘AUDPC’, respectively, were used as fixed factors. The results of our analysis yielded a non-significant effect of the ‘Year’ and ‘Biological Replicate’ factors on both PDI *(p* = 0.363) (Table S6) and AUDPC (*p* = 0.607) (Table S7) values, confirming the reproducibility of the observations conducted independently in 2018 and 2019.

### 2.4. Effects of P. indica Treatment on Onion Biochemical Response to Stemphylium Leaf Blight Disease

#### 2.4.1. Hydrogen Peroxide Content and Lipid Peroxidation

**Figure 4:**
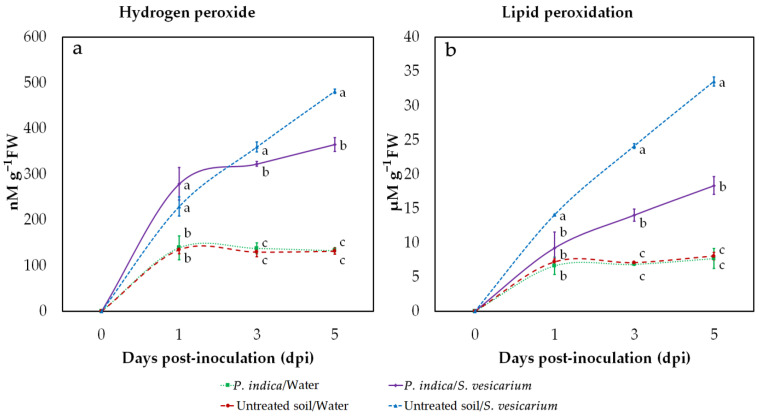
Effects of *P. indica* treatment and *S. vesicarium* infection on onion hydrogen peroxide content (**a**) and lipid peroxidation (=malondialdehyde content) (**b**). The enzymatic assays were performed at 1, 3, 5 days after *S. vesicarium* or mock inoculation. Values are expressed as the average of three biological replicates, each consisting of three plants pooled together. Statistically significant differences were assessed by one-way analysis of variance (ANOVA) followed by a post hoc Duncan’s multiple range test. Different letters indicate statistically significant differences (*p*-value < 0.05). Error bars indicate the standard error of the mean. Point interpolation was done in Microsoft Excel^®^ 2016, using the “smoothed line” function, based on a third-order Bézier Spline. FW: fresh weight.

Hydrogen peroxide (H_2_O_2_) and lipid peroxidation levels in onion leaves were measured at 1, 3, and 5 days after *S. vesicarium* or mock inoculation, in greenhouse conditions. Pathogen-infected plants showed a steady increase in H_2_O_2_ levels, already evident at 1 dpi. The co-inoculation with *P. indica* resulted in a significant reduction in this effect, effective at 3 dpi and more evident at 5 dpi (Figure 4a, Table S8). The same trend was observed for malondialdehyde (MDA) levels, a marker of lipid peroxidation. In this case, the attenuation of the relevant increase due to *S. vesicarium* infection, in plants treated with *P. indica*, was even more marked, with a significant decrease already measurable at 1 dpi, which reached its peak at 5 dpi (Figure 4b, Table S8).

However, in the absence of *S. vesicarium*, no increase in H_2_O_2_ content and lipid peroxidation, nor significant differences with untreated controls, were observed in plants treated with *P. indica*. This suggests that the pathogen was solely responsible for the increase in H_2_O_2_ and MDA levels and that *P. indica* was able to mitigate this effect (Figure 4a,b, Table S8).

#### 2.4.2. Activity of Antioxidant and Defence Enzymes

**Figure 5:**
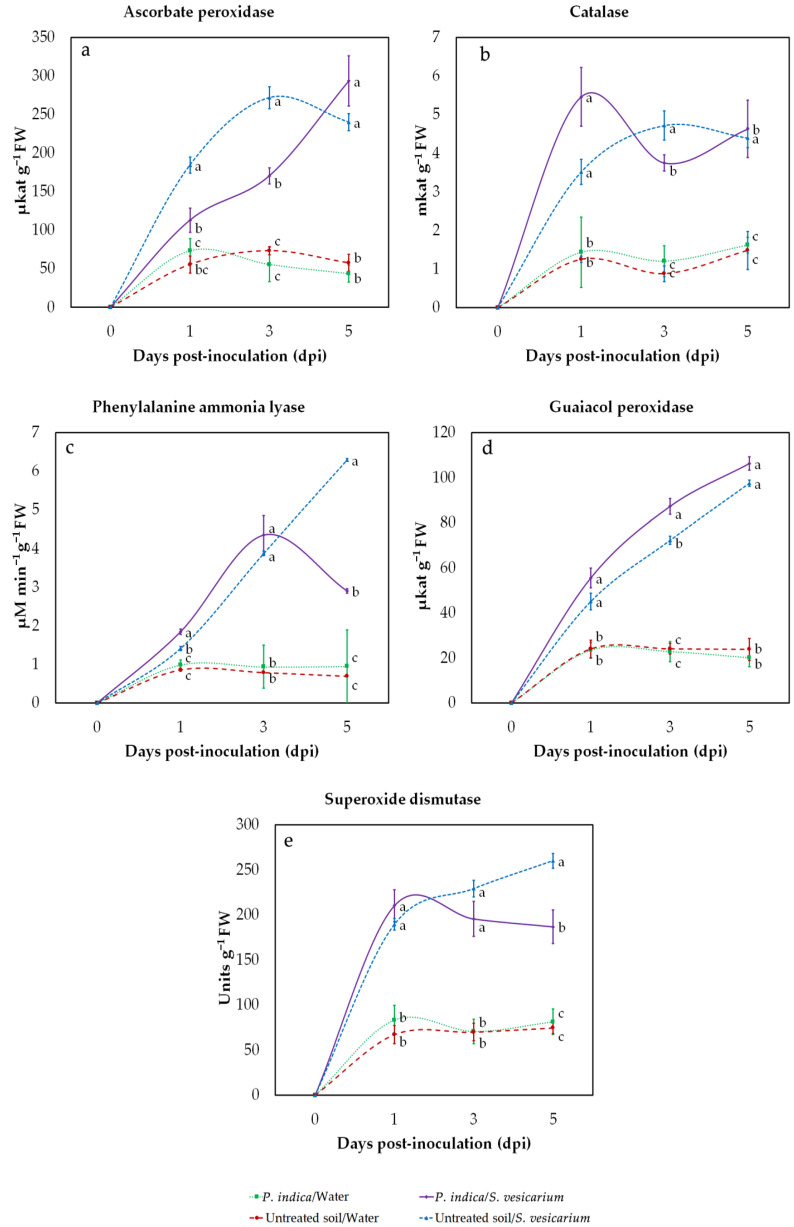
Effects of *P. indica* treatment and *S. vesicarium* infection on *A. cepa* antioxidant and defence enzymes. The enzymatic activity assays of ascorbate peroxidase (**a**), catalase (**b**), phenylalanine ammonia-lyase (**c**), guaiacol peroxidase (**d**) and superoxide dismutase (**e**) were performed at 1, 3, 5 days after *S. vesicarium* or mock inoculation. Values are expressed as the average of three biological replicates, each consisting of three plants pooled together. Statistically significant differences were assessed by one-way analysis of variance (ANOVA) followed by a post hoc Duncan’s multiple range test. Different letters indicate statistically significant differences (*p*-value < 0.05). Error bars indicate the standard error of the mean. Point interpolation was done in Microsoft Excel^®^ 2016, using the “smoothed line” function, based on a third-order Bézier Spline. FW: fresh weight.

To assess the performance of *P. indica* in modulating onion biochemical response to *S. vesicarium* infection, we measured the activity of a panel of enzymes known to play a role in the antioxidant defence system. For this purpose, activity assays were conducted on leaves sampled at 1, 3, 5 days after *S. vesicarium* or mock inoculation, under greenhouse conditions. As can be seen from the plots in Figure 5, infection with *S. vesicarium* triggered a significant overall increase in the activity of the antioxidative enzymes we tested, which, on average, became more evident with the time course and the progression of SLB symptoms.

Plants challenged with the pathogen, without previous treatment with *P. indica*, showed an increase in enzymatic activity that followed a rapid and linear progression in the case of catalase (CAT) (Figure 5b), guaiacol peroxidase (GPX) (Figure 5d), and phenylalanine ammonia-lyase (PAL) (Figure 5c), whereas it was slower in the case of superoxide dismutase (SOD) (Figure 5e), or even decreased in the step from 3 to 5 dpi, in the case of ascorbate peroxidase (AXP) (Figure 5a).

What stood out in our analysis was the role of *P. indica* in interfering with the increased enzyme activities induced by *S. vesicarium*. However, it was not possible to describe an unambiguous trend, because the interaction between the pathogen and the biocontrol agent produced different effects in relation to different assays and time points. With regard to the short-term response, measured at 1 dpi, we observed that treatment with *P. indica* resulted in a synergistic increase in enzymatic activities, most noticeable in CAT (+55.11%) (Figure 5d), although it was statistically significant only in the case of PAL (Figure 5c, Table S8). A pronounced effect, of the opposite sign, was measured in the case of AXP (Figure 5a).

At 3 days after inoculation, infected plants treated with *P. indica* had reduced activity compared with untreated infected controls, in the case of SOD (−14.7%) (Figure 5e), CAT (−20.55%) (Figure 5b), and especially AXP (−37.27%) (Figure 5a). In the case of PAL (Figure 5c) and GPX (Figure 5d), however, activity remained higher in *P. indica*-treated plants.

The strongest effects could be observed at 5 dpi. At this time point, the effect of *P. indica* on the activity of PAL (−53.74%) (Figure 5c) and SOD (−28.13%) (Figure 5e) enzymes started to decrease and showed significant reductions compared to untreated controls. On the contrary, in treated plants, we measured a slight increase in the activity of CAT (+5.23%) (Figure 5b) and of the peroxidases AXP (+22. 31%) (Figure 5a) and GPX (+8.99%) (Figure 5d).

It is worth mentioning that none of the enzymes considered showed significant changes in activity in plants not infected by *S. vesicarium*, both treated and untreated with *P. indica*, except for a slight increase in AXP at 1 dpi, following treatment (Figure 5, Table S8). This corroborates the idea that *P. indica* is not likely to affect the defence enzyme response by itself, but has a role in modulating onion biochemical reaction to *S. vesicarium* infection.

### 2.5. Effects of P. indica Treatment and S. vesicarium Infection on Plant Defence-Related Gene Expression

To provide further evidence for the existence of an effect of *P. indica* treatment on host defence mechanisms against *S. vesicarium* infection, we collected leaves 5 days after *S. vesicarium* or mock inoculation, from onion plants grown in greenhouse conditions. Samples were used to perform a qRT-PCR expression analysis on seven genes involved in the plant defence pathway.

The experiment was repeated twice, relying on two independent sets of leaf samples, as described in Section 4.7.2. However, linear model analysis allowed us to exclude a significant random effect on gene expression due to the ‘Experimental replicate’ factor (*p* = 0.867, Table S9), so here we report representative data from one of the two repetitions.

**Figure 6:**
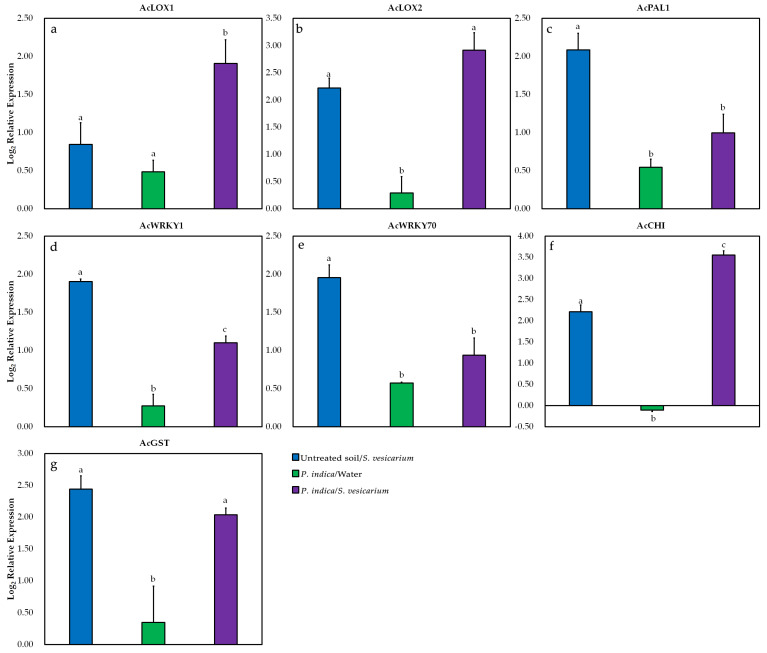
Effects of *P. indica* treatment and *S. vesicarium* infection on *A. cepa* defence-related genes. Relative expression of lipoxygenase 1 (*AcLOX1*) (**a**), lipoxygenase 2 (*AcLOX2*) (**b**), phenylalanine ammonia-lyase 1 (*AcPAL1*) (**c**), WRKY transcription factor 1 (*AcWRKY1*) (**d**), WRKY transcription factor 70 (*AcWRKY70*) (**e**), chitinase (*AcCHI*) (**f**), glutathione S-transferase (*AcGST*) (**g**) genes was evaluated at 5 days after *S. vesicarium* or mock inoculation and compared between *P. indica*-treated and untreated plants. Data refer to the most representative of two independent repeated experiments, which did not share statistically significant differences, as assessed by linear model analysis (lm). Each sample was collected from five plants pooled together. Each reaction was performed in triplicate wells, and values are expressed as the average of these three technical replicates. Statistically significant differences were assessed by one-way analysis of variance (ANOVA) followed by a post hoc Duncan’s multiple range test. Different letters indicate statistically significant differences (*p*-value < 0.05). Error bars indicate the standard error of the mean. Gene expression is calculated relative to the control treatment (untreated soil/water), whose value is set to log_2_ (1) and not shown.

Figure 1 shows that the presence of both *S. vesicarium* and *P. indica*, in whatever combination, generally increased the transcript levels of the selected genes, relative to the control plants (untreated soil/water). The only exception was the gene for chitinase (*AcCHI*) (Figure 6f), which was underexpressed (−0.11 log_2_ fold change, FC) in *P. indica*-treated and mock-inoculated plants.

Relevant increases in the relative expression levels of onion genes coding for WRKY transcription factor 1 (*AcWRKY1*) (+1.90 log_2_ FC) (Figure 6d), WRKY transcription factor 70 (*AcWRKY70*) (+1.95 log_2_ FC) (Figure 6e), phenylalanine ammonia-lyase 1 (*AcPAL1*) (+2.08 log_2_ FC) (Figure 6c), glutathione S-transferase (*AcGST*) (+2.44 log_2_ FC) (Figure 6g), lipoxygenase 2 (*AcLOX2*) (+2.22 log_2_ FC) (Figure 6b) and *AcCHI* (Figure 6f) (+2.21 log_2_ FC) were measured in *S. vesicarium*-infected plants in the absence of *P. indica* treatment, and thus solely attributable to the presence of the pathogen and the emergence of SLB disease.

Gene encoding for lipoxygenase 1 (*AcLOX1*) (Figure 6a) showed less pronounced relative increases in infected plants not treated with *P. indica* (+0.84 log_2_ FC), whereas up-regulation of the transcript levels of this gene was more pronounced in plants infected and treated with *P. indica* (+1.91 log_2_ FC). This remarkable and significant increment has been measured in the same plants for Ac*LOX2* (+2.91 log_2_ FC) (Figure 6b) and Ac*CHI* (+3.55 log_2_ FC) (Figure 6f).

As a general observation, in the case of *AcLOX1* (Figure 6a), *AcCHI* (Figure 6f) and, to a lesser extent, *AcLOX2* (Figure 6b) genes, the simultaneous presence of *P. indica* seemed to mask the induction of an infection-driven response, which, instead, remained much more evident for transcription factors *AcWRKY1* (Figure 6d) and *AcWRKY70* (Figure 6e), *AcPAL1* (Figure 6c) and *AcGST* (Figure 6g).

In the absence of infection, the presence of *P. indica* alone did not cause remarkable increases in the transcription levels of the onion genes we selected. Relative expression remained bounded in a range between 0.29 and 0.57 log_2_ FC for almost all genes, apart from *AcCHI*, which, as mentioned, was downregulated (Figure 6, Table S10). This seems to demonstrate that, as observed in relation to the enzymatic response, the induction of the defence reaction by the plant is triggered by the pathogen, but, at the same time, *P. indica* exerts a role in modulating this response, more or less evident depending on the pathways considered.

### 2.6. Statistical Analysis of the Effects of P. indica Treatment on Plant Response Variables to S. vesicarium Infection

**Figure 7:**
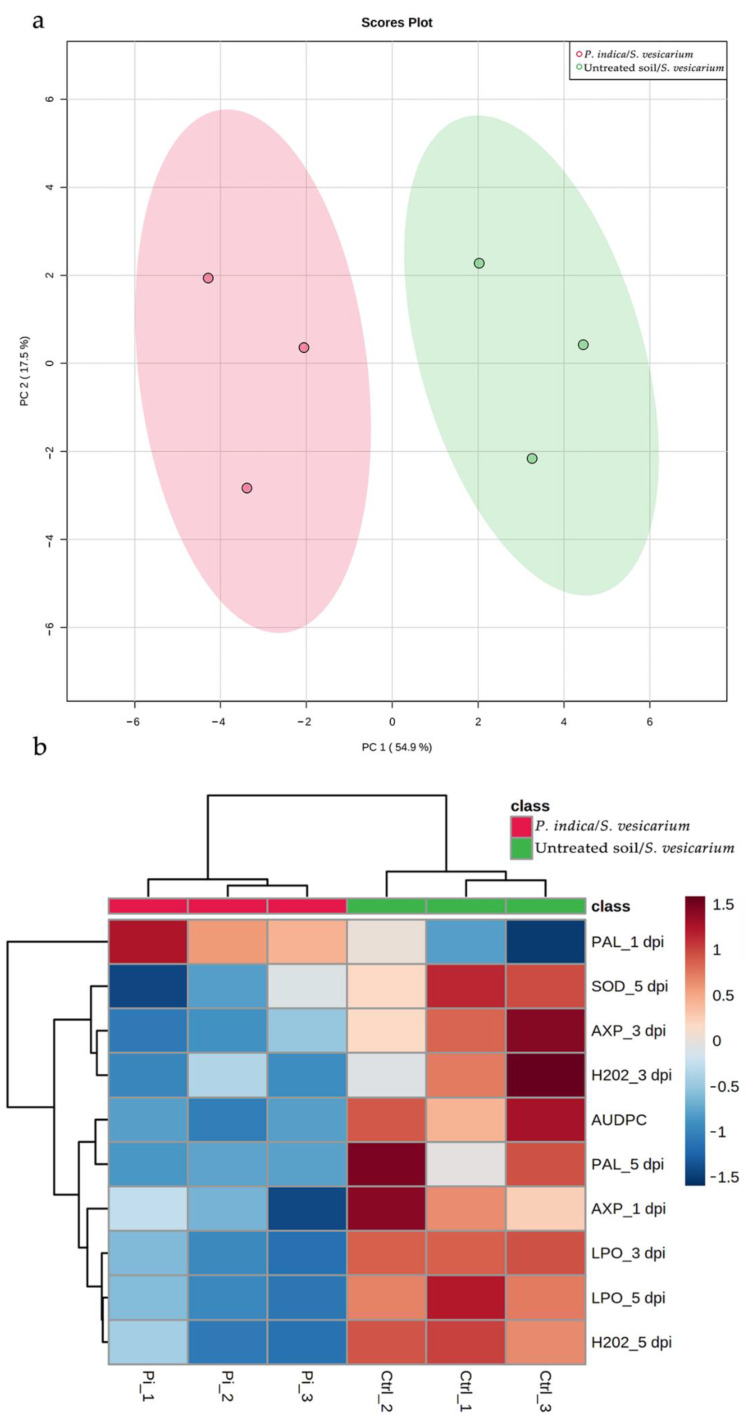
Principal component analysis (PCA) (**a**) and hierarchical clustering analysis (HCA) heatmap (**b**) of individual onion response variables to *P. indica* treatment and *S. vesicarium* infection, in greenhouse conditions.Data relative to percent disease index (PDI), area under disease progress curve (AUDPC) and enzymatic assays, collected at 1, 3, 5 days after *S. vesicarium* inoculation, were used in both analyses to compare *P. indica*-treated and untreated plants. For scaling, data were mean-centred and divided by the standard deviation of each variable. Score plots between the selected principal components (**a**) show the explained variances in brackets. Each point in the plot represents a biological replicate. Hierarchical clustering was performed based on Euclidean distance and Ward’s clustering algorithm. HCA results are shown as a heatmap diagram (**b**) where rows represent the response variables and columns the individual biological replicates. For clarity, only the ten highest-ranked variables out of the twenty-five included in the analysis are shown. PAL: phenylalanine ammonia-lyase; SOD: superoxide dismutase; AXP: ascorbate peroxidase; LPO: lipid peroxidation; dpi: days post-inoculation.

Principal component analysis (PCA) was performed to allow an unbiased comparison of onion response to *S. vesicarium* infection, in the presence or absence of treatment with *P. indica*. For this purpose, twenty-five of the parameters described above, and measured under greenhouse conditions, were included in the analysis. In the PCA model we derived, the first two principal components accounted for 72.4% of the total variance (PC1: 54.9%, PC2: 17.5%). As shown in Figure 7a, the score plot of the two principal components clearly separated samples treated with *P. indica* before *S. vesicarium* inoculation, from untreated controls.

The same dataset of plant response variables was used for a hierarchical clustering analysis (HCA) heatmap. In agreement with PCA results, *S. vesicarium*-infected plants treated with *P. indica* were clustered together and distinct from those challenged with the pathogen, without any treatment (Figure 7b). To retain the most contrasting patterns between the two groups we identified the ten most significantly divergent features. Only one of these variables (PAL activity at 1 dpi) increased considerably in the treated plants and formed a separate cluster in the HCA-associated dendrogram. In contrast, the other nine variables, which clustered together, showed higher values in untreated plants, while exhibiting a marked reduction due to treatment with *P. indica*. In addition to a disease marker represented by AUDPC value, this cluster largely consisted of the biochemical changes measured at 5 dpi (PAL and SDO activity, H_2_O_2_ content, lipid peroxidation).

According to these data, we can infer that the onion biochemical response becomes, as was to be expected, more relevant as the *S. vesicarium*-induced disease progresses, and foliar damage worsens. The efficacy of treatment with *P. indica*, in turn, appears to be manifest and interacts on several biochemical parameters, which, as our statistical analyses demonstrate, are interrelated.

## 3. Discussion

In this study, we have evaluated the efficacy of the beneficial fungal endophyte *P. indica* against the harmful SLB disease associated with S. *vesicarium* infection in onions. In our effort, we have also been interested in providing an initial and overall assessment of biochemical and molecular markers that could prove the indirect effect elicited by *P. indica* on the response of onion to the infection by *S. vesicarium*.

Our results proved that *P. indica* treatment was able to boost plant growth parameters, stimulating leaf length and root biomass. This growth-promoting effect of *P. indica* has been shown in other crops and several species from different plant families [36,38,52]. Several hypotheses have been formulated so far about the mechanisms of action of the fungus that may interact with the promotion of vegetative growth [36]. The relevant growth increase that we observed, following treatment, in both leaf portion and root biomass, suggests that also in onion, as reported in barley [49] and Chinese cabbage (*Brassica campestris* subsp. *chinensis*) [53], *P. indica* may actively promote auxin-regulated genes through the production of indole-3-acetic acid-like compounds. However, at the current stage of knowledge, other ways of action cannot be excluded. In fact, it has been demonstrated that *P. indica*, like many rhizosphere colonisers, is capable of hijacking ethylene signalling [54]. Since, under natural conditions, ethylene is employed by the plant to inhibit its growth, alterations in the ethylene pathway caused by *P. indica*, in turn, may help promote host growth [55]. Lacking more targeted research, however, we cannot exclude that *P. indica*-colonised onions might not have also benefited from improved nutrient uptake, as in the case of some legume species [56]. However, even if this were demonstrated, it would remain an open question whether *P. indica* facilitates the uptake of nutrient elements and thus the plant grows faster, or whether, instead, by promoting root growth, the fungus directly favours the absorption of minerals from the soil [36].

The results of our investigations clearly showed that *P. indica* has the potential to be used as a biocontrol agent against SLB onion disease. We were able to prove that treatment with the endophytic fungus is effective in significantly reducing disease symptoms elicited by the pathogen. Although it was easier to measure this effect under greenhouse conditions, where it was of greater magnitude, encouraging and well-established results were also obtained in field trials, repeated in two different seasons. The relatively lower efficacy of the treatment on disease progression in an open-field environment may have been biased by the high density of *S. vesicarium* inoculum, naturally occurring, especially in 2018. In any case, it is well known that, unlike pot experiments, which are performed under soil-controlled conditions, the action of the beneficial microorganism, under field conditions, is modulated by an intricate interaction with soil microbial networks [57]. This type of interaction gains relevance in the case of *P. indica*, whose efficacy as a biocontrol agent has been proven to be significantly influenced by the relationships it establishes with the microbial communities coexisting in the rhizosphere and root endosphere [34,52].

The effectiveness of *P. indica* in controlling SLB disease does not come unexpected, as in recent years, extensive data have been collected on its biocontrol potential against a plethora of foliar and root pathogens [37]. Many recent studies have shown that, following *P. indica*-treatment, several crop species may acquire resistance against leaf powdery mildew caused by *Blumeria graminis*, root rot caused by *Cochliobolus sativus* [41], *Fusarium graminearum* [58], *F. culmorum* [42], *Rhizoctonia solani* [34], Verticillium wilt caused by *Verticillium dahliae* [38] and many other diseases, frequently added to an ever-growing list. Despite this extensive research effort targeting several crops of agronomic interest, to our knowledge, the possibility of exploiting *P. indica* for the control of SLB, as well as other soil-borne fungal diseases affecting onion and garlic, had never been investigated.

In our field evaluation, we compared the efficacy of treatment with *P. indica*, with that of the most common commercial fungicides used to control SLB disease in onion fields. It should be noted that, as was to be expected, *P. indica* could not exert a reduction of the disease comparable in entity with the results we obtained following treatment with the synthetic fungicides we tested. However, as we observed that *P. indica* significantly delays the progress and reduces the extent of the disease, we believe that the integrated use of fungicides with the periodical release of *P. indica* could minimise the frequency and the volume of chemical spray applications. What remains to be ascertained is the tolerance of the biocontrol agent to the simultaneous release of doses, albeit limited, of the selected fungicide. Once established that the survival rate of the beneficial microorganism is not heavily affected by the action of the targeted fungicide, this type of integrated approach is now widely used in many pathosystems [59]. Synergistic application of fungicides and fungicide-tolerant biocontrol agents was successfully used in the management of several fungal diseases, such as Botrytis gray mold of grapes [60], Fusarium wilt disease in dry bean [61], rosemary Rhizoctonia aerial blight [62], Fusarium crown and root rot in tomato [63], Botrytis blight in ornamental plants [64]. Even more sustainable integrated approaches could be introduced to completely replace fungicide sprays.

The new frontier in the application of *P. indica* and other beneficial biocontrol agents, which completely bypasses the risk that combined use with fungicides may impact their survival rate in the root system, is based on synergy with other beneficial microorganisms [65]. Recent findings suggest that several bacterial strains can stimulate *P. indica* growth and may boost beneficial effects when jointly applied with the fungus [34]. Interesting, beneficial effects for mung bean (*Vigna mungo*) growth were obtained from the synergy between *P. indica* and the fluorescent *Pseudomonas* sp. R81 [56]. Co-inoculation of *P. indica* and bacterial strains of the genus *Mycolicibacterium*, even showed that this microbial consortium is able to ease the severe damages induced by *F. oxysporum* and *R. solani* in tomato [34]. It is true that in some instances the interaction with other microbial species can exert an antagonistic effect on the performance of *P. indica* [66], and a long way awaits to be done, towards the knowledge of the mechanisms that regulate these combinations. Most interestingly, Anith et al. [65] successfully explored how the sequential application of *P. indica* and the highly efficient antagonist *Trichoderma harzianum*, can be highly effective on black pepper (*Piper nigrum* L.) growth and in countering *Phytophthora capsici* infections. This is relevant for our purposes because the few reported attempts before our study to implement biological control of *S. vesicarium* in onion have relied on *Trichoderma* species [13,30]. Therefore, selection within the rhizosphere of beneficial bacteria or *Trichoderma* spp. strains compatible with *P. indica* and effective in increasing its biocontrol action against *S. vesicarium* in onion may be a worthwhile endeavour.

Although it is well known that colonisation by *P. indica* can protect a wide range of plant species from fungal pathogens, there is not a clear consensus on its mechanism of action. However, we know that *S. vesicarium* is generally considered a secondary pathogen that attacks already damaged onion leaf tissues, and thus a full-fledged leaf pathogen [13]. Research has reached a consensus in claiming that, against foliar pathogens, *P. indica* exerts its effects by inducing systemic resistance, analogous to the well-characterised mechanisms described for plant growth-promoting rhizobacteria (PGPR) [67,68]. Typically, these mechanisms of ISR are reflected in the up-regulation of genes involved in defence pathways [49,69] and overt biochemical responses involving, among others, reactive oxygen species (ROS) and metabolites associated with phenolic pathways [70].

Based on this awareness, we have been particularly interested in finding some insights that might suggest that the effects of *P. indica* against SLB disease that we had observed might trace their origins to the induction of a biochemical and molecular response in the plant.

Plants under biotic stress are well known to produce ROS which play an important role in limiting invasion of obligate pathogens, through the induction of hypersensitive response and programmed cell death [71,72]. However, as the process of pathogenesis progresses, the involvement of ROS can result in deleterious effects, and foster the establishment of necrotrophic pathogens [73]. To counteract the detrimental effects of the overproduction of ROS generated during the response to pathogens, plants are usually equipped with a complex panel of enzymatic and non-enzymatic antioxidants [73,74]. Alterations in the levels of these biochemical markers may be indicative of an overproduction of ROS, as a result of the pathogen attack, and the consequent plant defence response, even in cases where it is triggered by beneficial microorganisms.

In our greenhouse experiments, we observed that infection with *S. vesicarium* resulted in a rapid increase in the content of malondialdehyde (MDA), whose formation is known to be a marker of peroxidative damage to lipid membranes. However, this effect was significantly reduced in *P. indica*-colonised plants. Inhibition of MDA accumulation would seem a sign of reduced lipid peroxidation, and this enhanced protection against ROS damage was also shown in *Trichoderma harzianum*-inoculated sunflowers (*Helianthus annuus*) challenged with *R. solani* [75].

Lipid peroxidation, a sign of oxidative stress induced by *S. vesicarium*, was coupled, in our experience, with a significant progressive increase of H_2_O_2_ levels. As mentioned in general for ROS, H_2_O_2_ has wide involvement in plant stress tolerance and disease resistance and plays an acknowledged role in constraining fungal pathogen growth [76,77]. On the other hand, it is important that plants scavenge H_2_O_2_ excess, as its increased level may lead to cell damage [78]. What came from our observations is that H_2_O_2_ level in *P. indica*-colonised infected plants was higher at an early stage of infection which could be acting as a signal of oxidative stress [79], as H_2_O_2_ has been shown to play also an essential role in signal transduction [78]. The observation that *P. indica* may contribute together with the pathogen to increase H_2_O_2_ concentration in leaves in the early stages of infection, as a mechanism of induced resistance, had already been reported in chickpea (*Cicer arietinum*) plants infected by *Botrytis cinerea* [80] and wheat challenged with *Blumeria graminis f. sp. tritici* [42].

As could be expected, onion plants responded to the biotic stress induced by *S. vesicarium*, with a rapid and significant increase in the activity of enzymes responsible for antioxidative protection and involved in the phenylpropanoid pathway. What we found interesting was to reveal how *P. indica* was able to modulate, and in some cases elicit at higher levels, these activities, especially in the early stages of infection.

In our greenhouse study, we observed that the activity of SOD, GPX, PAL, and CAT enzymes was even more increased in plants in which infection with *S. vesicarium* was preceded by treatment with *P. indica*. This remarkable effect was observed especially at 1 dpi, i.e., in the early stages of infection, and then tended to decrease as the infection progressed. In contrast, we observed a constant increase over time only in the case of APX and GPX peroxidases.

The priming effect of SOD activity in the initial phases of infection is a clue to the possible induction of ISR, as it was demonstrated in many PGPR, providing analogous protection against several fungal and bacterial pathogens [81]. SOD is a multimeric metalloenzyme, and its prompt activation represents a crucial step in the first line of plant defence, as it catalyzes the dismutation of superoxide radicals into the less toxic H_2_O_2_ and O_2_, fundamental to prevent oxidative damage [82]. Often SOD enzymes play this barrier role against pathogen attack in conjunction with peroxidases [73]. In particular, GPX, apart from neutralizing free radical generation, plays an active role as an antifungal defence enzyme [83]. In fact, through the oxidation of phenolic compounds, such as guaiacol (o-methoxyphenol), this enzyme contributes to providing the plant with basic metabolites required to synthesise lignins and other structural barriers that prevent the pathogen from spreading into tissues [84,85]. The fact that *P. indica* may be able to modulate an early response associated, in the first hours after infection, with increased activity for GPX, but also for PAL, another enzyme primarily involved in the activation of the phenylpropanoid pathway, is not surprising. PAL and GPX are among the most well-characterised biochemical markers of induced resistance [86,87], and their synergistic activation in the early phases of infection, has often been associated with treatment with beneficial microorganisms or even microbial consortia [73,88]. The specific trend we observed in the activity of CAT and AXP in plants treated with *P. indica* can be explained in relation to the fluctuations in the levels of H_2_O_2_ we also observed. CAT and AXP are, indeed, the most active scavengers of H_2_O_2_ available to the plant antioxidant defense system. However, CAT fulfils its function in the presence of bulk concentrations of H_2_O_2_, while AXP, relying on higher affinity for the oxidizing substrate, and a more widespread presence in several subcellular compartments, can also take effect at lower concentrations [89,90]. This is exactly what may have occurred in our case, due to *P. indica*-derived modulation, which primed an initial boosting of CAT activity in the early stages of infection, when the level of H_2_O_2_ was highest, and then resulted in increased AXP activity when H_2_O_2_ became less accessible. This shift between catalytic and peroxidase activity, as the defence response induced in the plant progresses, had already been reported in tomato plants affected by early blight disease, caused by *Alternaria solani* [91].

At the molecular level, to better support the evidence that *P. indica* was capable of triggering and enhancing the onion response induced by *S. vesicarium* infection, we have analyzed, at a late stage of infection, the relative expression of seven genes known to be involved in plant defense. What we found in our observations was that the simultaneous presence of *P. indica* and the pathogen led to a significant upregulation of *AcLOX1* and *AcLOX2* lipoxygenases, and of *AcCHI* chitinase.

Increased mRNA levels of multiple *LOX* isoforms have been reported for several pathosystems and correlated with induced plant defence responses [92,93,94]. Genes coding for *LOX* dioxygenases govern the catalytic hydroperoxidation of polyunsaturated fatty acid substrates, which in the case of higher plants are most commonly linolenic and linoleic acids [95]. Having often been associated with the occurrence of a hypersensitive response (HR) that constrains the infection site [96], the increase in *LOX* activity has been, in several cases, proven to be transcriptionally related to pathogen elicitors [92,97]. However, recent investigations have led to the discovery that heightened expression of defence-related lipoxygenases may be driven by microbe-associated molecular patterns (MAMPs) triggered by beneficial microbes [98]. MAMP-regulated activation of *LOX* genes may therefore be induced by signal molecules or secondary metabolites secreted by fungal and bacterial microbes, often inhabiting the rhizosphere [99]. Similar to what we observed in infected plants treated with *P. indica*, Cawoy et al. [100] have noted a significant increase in *LOX* expression on tomato plants challenged with *B. cinerea* and treated with *Bacillus amyloliquefaciens* strain S499. Interestingly, they showed that *LOX* expression was elicited when a lipopeptide, surfactin, was secreted by *B. amyloliquefaciens* S499, and this, in turn, triggered ISR.

If we assume that *P. indica* can rely on MAMPs recognisable by the onion plant receptors, considerable future effort must be devoted to identifying possible signal molecules. It is reasonable to believe that a possible *P. indica*-triggered ISR may involve jasmonate/ethylene (JA/ET) or salicylic acid (SA) signalling pathways [101]. Mobilisation of signal molecules potentially originating from the beneficial microbe, especially if they are secreted into the roots and are expected to reach the aerial parts, would require the activation of JA-, ET- or SA-dependent pathways [98,102]. The increased expression of *AcLOX1* and *AcLOX2* we observed, following *P. indica* treatment, may support this hypothesis, as several studies provided evidence that LOX genes play a prominent role in the regulation of both SA and JA pathways, and their modulation may be critical in controlling the balance of SA/JA signalling, and the consequent expression of ISR [103,104,105,106]. Although the activation of effective plant defence responses is often resulting from a cross-communication between SA and JA pathways, we should not be tempted to oversimplify the mechanism by which *P. indica* and *S. vesicarium* may have been involved in this crosstalk. Plant hormone signalling networks are far to be completely elucidated, especially under multi-fungal interactions, as in our case [106,107].

The effect of *P. indica* treatment prior to *S. vesicarium* infection was also relevant to the expression levels of *AcCHI*. Basic and acidic chitinases belong to the glycosyl hydrolase family and are involved in plant disease resistance mechanisms [108]. Due to their role in fungal cell wall degradation, they are included among the pathogenesis-related (PR) proteins and are significantly induced in response to necrotrophic fungal infections [109,110]. Similar to what we observed in *S. vesicarium*-infected onions, in a precedent study [44] we had shown that *P. indica* was responsible for an enhanced expression of *Solanum lycopersicum* chitinases, in the late stages of *A. solani* infection. Daneshkhah et al. [110] also confirmed that *P. indica*-mediated upregulation of PR chitinases could hardly be noticed in *A. thaliana* roots in the early stages of infection, during the biotrophic colonisation phase, and was expressed later in time, at the cell death-associated stage. As suggested by Van Wees et al. [67] chitinase regulation is primarily driven by ET/JA signalling pathway. Increasing evidence [110,111] also suggest that the effect of *P. indica* on chitinases is also intimately related to the action of hormonal signals. It is also possible that, as has been proposed [15], JA signalling may act in the early stages of *P. indica* colonisation, to weaken the defence responses associated with chitinase activity and favour root colonisation. Down-regulation of plant defence genes at early stages has been found to be a key factor in *P. indica* root colonisation [53,111,112]. This observation would also find support in the down-regulation of the *AcCHI* gene that we detected in response to colonisation by *P. indica*, in plants that were not inoculated with the pathogen. Nevertheless, upregulation of chitinase genes at an advanced stage of pathogen infection, may not only be related to plant defence response, but also hide their hitherto unknown role in *P. indica*-plant interaction. The hypothesis that chitinases, like other PR genes, may be involved in a range of functions, not only restricted to defence response, is gaining recent consensus [110,113], however, these processes are yet to be investigated.

In our trial, we could also observe transcriptional changes in other genes involved at a different extent in the regulation of SA- or JA-dependent defence pathways, but these could be solely attributed to *S. vesicarium* infection process, as they were most evident in plants not treated with *P. indica*. *PAL* gene has an important role in secondary metabolism and is involved in the initial steps of the phenylpropanoid pathway, leading to the biosynthesis of phenolic compounds involved in plant protection against pathogens [114,115]. The increased transcription levels of the *AcPAL1* gene we observed in coincidence with the progress of *S. vesicarium* infection was not surprising, as the upregulation of *PAL* is a well-known plant response to pathogen infection [116] that is often associated with systemic resistance [117]. The biosynthesis of SA is closely tied to *PAL* enzymatic activity, so this gene is often referred to as a marker of SA-dependent signalling and plays a pivotal role in systemic resistance [118,119]. Kim and Hwang [120] provided strong evidence on the role of plant immune response, as they showed that disruption of *CaPAL1* gene in *Capsicum annuum* led to enhanced vulnerability to *Xanthomonas campestris* pv. *vesicatoria* (Xcv), while its overexpression in transformed *A. thaliana* plants increased SA levels and conferred strengthened resistance to *Pseudomonas syringae* pv. *tomato* (Pst). Interestingly, it’s worthy to note that we measured *AcPAL1* expression levels at an advanced stage of infection. Coherently with the declining trend observed in the enzyme activity assay, we were able to confirm that the effects of *P. indica* on this gene appear to be mainly related to an early response, while its upregulation maybe later attributable to the infection progress and the establishment of defence response to *S. vesicarium* colonisation.

We have also included in our analysis *WRKY* factors, as these DNA-binding transcriptional regulators have been widely reported to be involved in plant disease resistance [121,122], induction of defence-related genes [123] and more in general to stress tolerance [124]. In a previous study on tomato plants, we had shown that *P. indica* colonisation could contribute to an upregulation of WRKY genes, which in turn positively regulated the expression of PR genes, thus suggesting the induction of resistance mechanisms against early blight disease. In the present study, we have found that *AcWRKY1* and *AcWRKY70* genes were upregulated in *S. vesicarium*-infected plants, however, this overexpression became less relevant when plants were also pre-treated with *P. indica*. The role of *WRKY70* gene in plant defence response has proved very intriguing and has generated a great deal of interest in recent years [125]. Li et al. [126] have reported that this transcriptional factor is able to induce expression of SA-related genes, while down-regulating JA-responsive pathways. In their studies on *A. thaliana*, they have proposed that *WRKY70* may represent a hub of coordination balancing SA- and JA-signalling and crucial for resistance to pathogen attacks. They proved that this gene could govern two distinct defence responses, as they observed that its over-expression caused enhanced SA-regulated resistance to *Erysiphe cichoracearum* and weakened the JA-mediated response to *Alternaria brassicola*, while impairing its function resulted in the opposite behaviour. *WRKY* fine-tuning of the balance between SA- and JA-dependent responses could be echoed in what we have observed, allowing us to hypothesise that differences in the expression of *WRKY* factors in response to infection, and in the presence/absence of *P. indica*, may imply that the latter impacts on their regulation and the downstream defence genes, with a cascade effect.

Taken together, our results on biochemical and molecular responses induced by *P. indica* in onion plants challenged with *S. vesicarium,* strongly emphasise the need to investigate the multiple interactions that the beneficial fungus may establish with the hormone signalling network. Interestingly, as it was observed in relation to *A. solani* early blight in tomato [127], treatment with *P. indica* alone could not induce significant upregulations of the genes we analysed. However, our data clearly shows that *P. indica* colonisation in onion tissues appears to enhance the systemic expression of the defence response triggered by the progression of *S. vesicarium* infection.

It also seems highly plausible that, as reported in other studies [128], host hormone signalling is hijacked by *P. indica* and this translates into the effects we observe on host defence machinery. However, although this sophisticated strategy of interaction with plant signalling is a well-established hypothesis and may also explain *P. indica* successful colonisation of a wide host range [128,129], current knowledge on its mode of action is very poor and focused studies should be strongly encouraged.

The complexity of the interaction of *P. indica* with the plant and its defence responses also lies in the symbioses that the fungus can establish with endocellular helper bacteria [37]. The best known of these symbiotic associations is the one it establishes with *Rhizobium radiobacter* strain F4 [50]. In our attempts, we did not microscopically observe that the spores and hyphae of *P. indica* were colonised by any bacterial cells, but more focused studies would be required, as these bacteria are known to be present in very low cell numbers, and are difficult to detect without specific molecular diagnostic probes [50,130]. However, it has been reported that among the metabolites secreted by *R. radiobacter* F4 in their symbiosis are N-acyl homoserine lactones important for the efficient colonisation of roots and plants endophytically [51]. These molecules, which together with diffusible signal factors (DSF) fatty acids are among the most common quorum-sensing regulators used by phytopathogenic bacteria, have themselves been found to be capable of inducing ISR in plants. It is not a stretch to think that molecules secreted by its endocellular symbionts may contribute to the effect exerted by *P. indica* [131]. Not to mention that, due to its biotrophic habit, the *P. indica* genome lacks genes coding for secondary metabolites, toxins and peptides, and that these additional defence elicitors could be supplemented by its symbiont bacteria [34].

From these considerations directly stems the need to direct subsequent research efforts into understanding how the potential application of *P. indica* in onion fields may alter or benefit resident soil communities. Evidence has already been gathered that the presence of *P. indica* is able to stimulate *Pseudomonas striata* populations in the rhizosphere [132] and may also directly influence the microbial communities associated with the root system of *Cicer arietinum* [133]. These interactions appear to be regulated by the ability of *P. indica* and other AMF-like microbes to establish an intimate relationship with root exudates [134] and to facilitate the access of pseudomonads and other PGPR to the apoplastic spaces of their plant hosts [135]. However, we cannot exclude that *P. indica* may also have a detrimental effect against some of the microorganisms inhabiting the onion rhizosphere, therefore culture-independent metagenomic studies of its overall impact on soil microbial ecology are further needed to support our promising results.

## 4. Materials and Methods

### 4.1. Plant Material and Fungal Culture

The highly SLB susceptible onion cultivar “Bhima Super” was used as plant material throughout this study. Pure culture of *S. vesicarium* was isolated from onions grown in the experimental field of Indian Council of Agricultural Research-Directorate of Onion and Garlic Research (ICAR-DOGR) located in Rajgurunagar (Pune, India) (18.8543° N, 73.8876° E). *S. vesicarium* culture was grown on potato dextrose agar (PDA) (HiMedia Laboratories, India) and incubated for 8–14 days at 28 ± 2 °C in a 16:8 h light/dark photocycle regime, to allow sporulation. The culture of *P. indica* was obtained from Dr J. Vadassery (National Institute for Plant Genome Research, New Delhi, India) and maintained on PDA medium. The cultivation and harvesting of *P. indica* mycelium used for inoculations were done as described in Roylawar et al. (2015) [44].

### 4.2. Assessment of P. indica Efficacy against Stemphylium Leaf Blight Disease

#### 4.2.1. Greenhouse Pot Experiment

Seeds of “Bhima Super” onion were sown in perforated plastic crates (50 × 33 × 28 cm, approximately) containing 10 g of minced *P. indica* mycelium per 100 g of autoclaved soil substrate. Control seeds were sown in autoclaved soil, in the absence of *P. indica*, using identical crates. Crates were kept in a climate-controlled greenhouse (28 ± 2 °C). After 45 days, onion seedlings were uprooted from the plastic crates and transplanted in pots (15 × 16 cm) containing soil-*P.indica* mixture and control seedlings in autoclaved soil. Plants were maintained in the same greenhouse conditions, following standardised cultivation practices, for further studies.

To assess whether *P. indica* had performed successful colonisation of onion roots by *P. indica*, roots of 10 randomly selected *P. indica*-treated plants were harvested at 45 days post-treatment and incubated at 90 °C in 10% KOH for 3 min, followed by 1 min in 1 N HCl, before staining with trypan blue (0.02%) for 60 min at room temperature. Root colonisation was observed under a DM2500 light microscope (Leica Microsystems, Wetzlar, Germany), and further confirmed by RT-PCR analysis carried out according to Lin et al. (2019) with *P. indica* specific primer pair *Tef*F (5′ TCCGTCGCGCACCATT 3′) and *Tef*R (5′ AAATCGCCCTCTTTCCACAA 3′), designed within the *Pitef1* gene coding for the transcription elongation factor 1α [136].

At 45 days after transplanting (DAT), *P. indica*-colonised and control plants were subjected to inoculation with *S. vesicarium.* Spore suspension of the pathogen was prepared by adding 5 mL of distilled water to the Petri dish and gently scraping the culture. The spore density was adjusted to 1 × 10^5^ mL^−1^ using an improved Neubauer haemocytometer (Rohem, India). Then, 1 mL of spore suspension was spread on onion leaves, while the same amount of distilled water was applied on the two series of plants not challenged with the pathogen, i.e., those exposed to *P. indica* colonisation, and those not-colonised. Plants were allowed to air-dry for 1 h, then sprayed with distilled water, covered with transparent plastic bags for 24 h and kept in greenhouse conditions at 28 ± 2 °C. The experiment was performed in triplicate, where one replicate consisted of a group of 10 pots for each of the four treatment combinations (*P. indica*/water, untreated soil/*S. vesicarium*, *P. indica*/*S. vesicarium*, untreated soil/water).

At 1, 3, and 5 days post-inoculation (dpi) damage induced by *S. vesicarium* on onion plants treated with *P. indica*, in comparison with untreated controls, was evaluated in terms of percent disease index (PDI). To this aim, SLB disease severity was recorded based on a five-point visual scale (0 = No disease symptoms; 1 = A few spots towards tip covering 10% leaf area; 2 = purplish-brown patches covering up to 20% leaf area; 3 = Several patches with paler outer zone covering up to 40% leaf area; 4 = Leaf streaks covering up to 75% leaf area; 5 = breaking of leaves from centre) [137]. PDI for each replicate pool (n = 10 plants) was calculated based on the following formula proposed by Wheeler [138].


$$PDI=\frac{Total\;number\;of\;ratings}{Number\;of\;observations\times Maximum\;grade\;in\;scale}\times 100$$

The area under disease progress curve (AUDPC) was calculated for each treatment combination using the R package *MESS* [139].

To allow further biochemical and gene expression analyses (see Section 4.4, Section 4.5, Section 4.6 and Section 4.7), leaf tissue was also collected at 1, 3, and 5 dpi, powdered in liquid nitrogen and stored at −80 °C for later processing. To this aim, three plants were randomly selected from those subjected to each of the four treatment combinations described above and pooled to constitute three biological replicates for treatment.

#### 4.2.2. Field Experiments

The field experiments were carried out at the experimental field of ICAR-DOGR in two consecutive *Kharif* crop seasons (from July to November) during the years 2018 and 2019. The average temperature ranged from 23 to 28 °C and the average humidity varied between 69 and 86%. The first part of the experiment was conducted under greenhouse conditions, and following a procedure identical to that described in the previous paragraph for the pot experiment. After 45 DAT, *P. indica*-colonised and untreated onion seedlings were uprooted from the perforated plastic crates and transplanted on raised beds (15 m^2^) in the open field, where they were exposed to *S. vesicarium* natural infection.

Biocontrol efficacy of *P. indica* was evaluated in comparison with three other fungicides commonly used to control SLB disease: Cabrio^®^ Top (Metiram 55% + Pyraclostrobin 5% WG) 2 g/L, Amistar^®^ Top (Azoxystrobin 20% + Difenoconazole 12.5%) 1.25 mL/L and Tilt^®^ (Propiconazole 25% EC) 1 mL/L. The foliar application of fungicides was done every 15 days till 60 DAT, on seedlings not treated with *P. indica*. Plants previously exposed to *P. indica* and controls were sprayed with distilled water only. The recommended package of agricultural practices was followed, except for fungicide sprays which varied with the treatments. The severity of SLB symptoms was recorded at 15, 30, 45, 60, and 75 DAT and expressed in terms of PDI, as previously described. The experiment was performed in triplicate, with one replicate consisting of a pool of 10 plants, according to a randomised block design (RBD), and repeated twice, as mentioned, in 2018 and 2019.

### 4.3. Measure of the Effects on Plant Growth Parameters

Vegetative growth parameters like leaf length, root length, root fresh weight, and root dry weight were measured at 60 DAT in control and *P. indica*-colonised plants kept under greenhouse conditions. Samples from three or five plants were collected to assess, respectively, leaf and root lengths, or root fresh and dry weights, and pooled to constitute three biological replicates for each treatment.

### 4.4. Evaluation of Hydrogen Peroxide Content

Hydrogen peroxide content was estimated according to the method described by Chavan et al. [140] with slight modifications. Briefly, 50 mg of each frozen leaf sample (see Section 4.2.1) was mixed in 500 μL of 4-(2-hydroxyethyl)-1-piperazineethanesulfonic acid (HEPES) buffer (50 mM; pH 7.5) and centrifuged for 5 min at 12,000 rpm (4 °C). The supernatant (100 μL) was mixed with 1 mL reaction volume containing 800 μL HEPES buffer (50 mM, pH 7.5) and 100 μL potassium titanium oxalate (2.5% in 20% H_2_SO_4_). Absorbance was recorded at 410 nm and H_2_O_2_ content was deduced from the standard calibration curve.

### 4.5. Measurement of Lipid Peroxidation

The lipid peroxidation was estimated as per Heath and Packer [141] with slight modifications. Powdered leaf tissue (0.5 g for each sample) stored at −70 °C (see Section 4.2.1) was homogenised in 5 mL of 0.1% trichloroacetic acid (TCA) and centrifuged at 12,000 rpm for 5 min at 4 °C. Four ml of 0.5% thiobarbituric acid (TBA) dissolved in 20% TCA were added to 1 mL of supernatant, then heated at 95 °C for 15 min, and rapidly cooled in ice. The mixture was centrifuged at 12,000 rpm for 10 min and absorbance of the supernatant was read at 532 nm and nonspecific absorbance at 600 nm was deducted. The malondialdehyde (MDA) content in the sample was determined by using an extinction coefficient of 155 mM^−1^ cm^−1^ [142].

### 4.6. Activity Assay of Antioxidant and Defence Enzymes in Leaves

#### 4.6.1. Preparation of Enzyme Extracts

Sampling was done as described earlier in Section 4.2.1. Powdered frozen leaf tissue (200 mg) from each sample was homogenised in 2 mL of ice-cold potassium phosphate buffer (0.1 M, pH 7.2) with 10 μL of ethylenediaminetetraacetic acid disodium salt (Na-EDTA) (0.1 M) and polyvinylpyrrolidone (0.5%). Homogenate was centrifuged for 20 min (20,000 rpm at 4 °C) and the supernatant was used for the estimation of enzymatic activities.

#### 4.6.2. Catalase

Catalase assay was performed according to [143]. The assay was carried out in a 3 mL reaction volume containing potassium phosphate buffer (0.1 M, pH 7.0), H_2_O_2_ (30 mM), and enzyme extract. The H_2_O_2_ oxidation was assayed at 240 nm every 30 s for 3 min, and activity was calculated using a molar extinction coefficient of 36 M^−1^ cm^−1^.

#### 4.6.3. Ascorbate Peroxidase

Ascorbate peroxidase activity was determined according to Nakano and Asada [144]. The reaction, performed in a 3 mL volume, contained a mixture of Tris- HCl buffer (0.5 M, pH 7.2), Na-EDTA (0.1 mM), ascorbic acid (0.5 mM), H_2_O_2_ (5 mM) and enzyme extract. The absorbance at 290 nm was monitored every 30 s for 3 min. AXP activity was determined using the molar extinction coefficient of 2.8 mM^−1^ cm^−1^.

#### 4.6.4. Guaiacol Peroxidase

Guaiacol peroxidase activity was measured as described by Xu et al. [66]. The assay was performed in a 3 mL reaction mixture containing phosphate buffer (0.1 M, pH 7.2), guaiacol (30 mM), H_2_O_2_ (20 mM), and enzyme extract. GPX activity was determined using the molar extinction coefficient of 26.6 mM^−1^ cm^−1^.

#### 4.6.5. Superoxide Dismutase

Superoxide dismutase activity was measured according to Beauchamp and Fridovich [145]. The assay was performed in a 3 mL reaction volume containing phosphate buffer (50 mM, pH 7.8), riboflavin (60 μM), methionine (20 mM), EDTA (1 mM), NBT (1 mM), and 70 µL enzyme extract. The reaction mixtures were prepared in two replicate sets for each treatment. One set was kept in the dark and the other was incubated in the light for 20 min under 100 μE m^−2^ s^−1^ light intensity at 26 ± 2 °C. Absorbance was measured at 560 nm, and the dark-incubated reaction mixtures of each sample were used as a blank.

#### 4.6.6. Phenylalanine Ammonia-Lyase

The activity of phenylalanine ammonia-lyase was measured as reported by Khan and Vaidyanathan [146] with slight modifications. For enzyme extraction, 1 g of each leaf sample was ground in liquid nitrogen and homogenised in 3 mL of cold Tris-HCl buffer (50 mM, pH 8.0), containing 2-mercaptoethanol (1.5 mM) and 0.5% polyvinylpyrrolidone (PVP), then centrifuged at 12,000 rpm for 10 min at 4 °C. The resultant supernatant was used as an enzyme extract for the PAL assay. The assay was performed in a 3 mL reaction mixture containing 1.5 mL of Tris-HCl buffer (50 mM, pH 8.0), 200 µL of L-phenylalanine (1.5 mM), 0.9 mL of distilled water, and 0.3 mL of enzyme extract. The reaction mixture was incubated at 30 ± 1 °C for 90 min, then 1 mL of HCl (2 N) was added to stop the reaction. Further, 3 mL of toluene were added to the mixture before centrifugation at 14,000 rpm for 10 min at 4 °C. The upper layer (enzyme-toluene mixture) was removed carefully and read at 290 nm. Toluene (in the absence of the enzyme) was used as a blank. A standard curve for trans-cinnamic acid (0–100 μM) was plotted to deduce the quantity of cinnamic acid formed in the sample.

### 4.7. Expression Analysis of Plant Defence-Related Genes

#### 4.7.1. Selection of Onion Target Genes

Expression analysis by quantitative Real-Time PCR (qRT-PCR) was performed, aiming to evaluate the differential response to the four treatments described in Section 4.2.1, of seven genes with a key role in plant defence reaction. To this aim, the following genes were included in our analysis: *A. cepa* lipoxygenase 1 (*AcLOX1*), *A. cepa* lipoxygenase 2 (*AcLOX2*), *A. cepa* chitinase (*AcCHI*), *A. cepa* glutathione S-transferase (*AcGST*), *A. cepa* phenylalanine ammonia-lyase 1 (*AcPAL1*), *A. cepa* WRKY transcription factor 1 (*AcWRKY*1) and *A. cepa* WRKY transcription factor 70 (*AcWRKY*70). *A. cepa* actin (*Ac*ACT) was used as a housekeeping gene. Primer design was performed using Gene Runner software (Hastings Software, Hastings, NY, USA), based on onion target sequences already available in the NCBI database (Table S11).

#### 4.7.2. RNA Isolation and cDNA Synthesis

Leaf samples collected at 5 dpi and stored at −80 °C, as previously reported, were used to perform total RNA extraction. Each sample consisted of leaves collected from a pool of five plants. Sampling of leaf pools was repeated twice to be used in two independent assessments. Following isolation using the RNeasy Plant Mini Kit (Qiagen, Hilden, Germany), total RNA was treated with DNase I (Thermo Fisher Scientific, Waltham, MA, USA), to remove potential genomic DNA contaminants. The RNA integrity was assessed by electrophoresis on a formaldehyde-agarose gel (1%) and the concentration was quantified using a NanoDrop 1000 spectrophotometer (Thermo Fisher Scientific, Waltham, MA, USA). Single-stranded cDNA was synthesised by reverse transcription from 1 μg of total RNA using RevertAid First Strand cDNA synthesis kit (Thermo Fisher Scientific, Waltham, MA, USA).

#### 4.7.3. qRT-PCR Analyses

The qRT-PCR reactions were carried out in a final volume of 10 μL containing 1 μL of cDNA, 1 µM of each primer, 2 μL of water and 5 μL of LightCycler^®^ 480 SYBR Green I Master mix (Roche Life Science, Penzberg, Germany). The thermal cycling profile was programmed as follows: initial denaturation at 95 °C for 5 min, 40 cycles of 95 °C for 15 s, 55–60 °C for 45 s, and 72 °C for 30 s. A melting curve analysis was performed from 65 °C to 95 °C with a 0.5 °C increase every 5 s, to confirm PCR specificity. Amplifications were run in a LightCycler^®^ 480 II System device (Roche Life Science, Penzberg, Germany). Each reaction was performed in triplicate wells, as to allow three technical replicates. Cycle threshold (CT) values resulting from qRT-PCR were normalised to the housekeeping gene (*AcACT*), and relative transcript abundance was calculated according to the 2^−∆∆Ct^ method [147], using the control treatment (untreated soil/water) as calibrator (fold change value set to 1). The experiment was repeated twice, on two independent sets of samples.

### 4.8. Statistical Analysis

Statistical analyses were performed using RStudio version 1.2.5033 [148] and the R packages *dplyr* [149], *agricolae* [150], *rstatix* [151], and *car* [152]. Data normal distribution and homogeneity of variances were assessed according to the Shapiro–Wilk’s and Levene’s tests, respectively [153]. Statistical significance of mean differences was evaluated by Student’s *t*-test or one-way analysis of variance (ANOVA), followed by post hoc pairwise comparisons using Duncan’s multiple range test. A linear model analysis (lm) was used to exclude the occurrence of statistically significant differences, due to random effects, between the two independent experiments of disease control in the field (Section 4.2.2) and gene expression analysis (Section 4.7).

Principal component analysis was carried out using MetaboAnalyst 5.0 platform [154] to compare plant response to SLB disease under greenhouse conditions, in the presence or absence of treatment with *P. indica*. To this aim, all data points relative to PDI, AUDPC, and enzymatic assays available for the greenhouse experiment, were included in the analysis. The same input dataset was used to evaluate similarities and contrasts between the response variables, in the two treatments (presence/absence of *P. indica*), through a hierarchical clustering analysis heatmap. HCA was also generated using MetaboAnalyst 5.0, referring to the Euclidean distance and Ward clustering algorithm. Before PCA and HCA data were submitted to auto-scaling (mean-centred and divided by the standard deviation of each variable).

For all statistical analyses, a *p*-value less than 0.05 was considered statistically significant. Graphic plots were created with Microsoft Excel^®^ 2016 spreadsheet (Microsoft Corporation, Redmond, WA, USA) or the R package *ggplot2* [155]. Adobe Photoshop^®^ CS6 software (Adobe Systems Inc., San Jose, CA, USA) was used to assemble and label the final figures.

## Data Availability

Data used for the aim of this study have been presented within the text and the Supplementary Materials. Raw data used for statistical analyses are available for further use on request from the corresponding author.

## Supplementary Materials

The following are available online at https://www.mdpi.com/article/10.3390/pathogens10091085/s1. Figure S1: Effects of *P. indica* treatment on onion growth parameters; Figure S2:Reduction of Stemphylium leaf blight disease symptoms on onion treated with *P. indica*, compared to the untreated control, as observed 5 days after inoculation with *S. vesicarium;* Table S1: Results of statistical analysis of the effects of *P. indica* treatment on onion growth parameters; Table S2: Results of statistical analysis of the effects of *P. indica* treatment on Stemphylium leaf blight PDI, in greenhouse conditions; Table S3: Results of ANOVA statistical analysis of the effects of *P. indica* treatment on Stemphylium leaf blight AUDPC, in greenhouse conditions; Table S4: Results of ANOVA statistical analysis of the effects of *P. indica* treatment on Stemphylium leaf blight PDI, in field conditions; Table S5: Results of ANOVA statistical analysis of the effects of *P. indica* treatment on Stemphylium leaf blight AUDPC, in field conditions; Table S6: Results of the linear model analysis of the factors affecting Stemphylium leaf blight PDI, in field conditions; Table S7: Results of the linear model analysis of the factors affecting Stemphylium leaf blight AUDPC, in field conditions; Table S8: Results of ANOVA statistical analysis of the effects of *P. indica* treatment and Stemphylium leaf blight disease on onion biochemical response; Table S9: Results of the linear model analysis of the factors affecting qRT-PCR expression analysis of onion defense-related genes; Table S10: Results of ANOVA statistical analysis of the effects of *P. indica* treatment and Stemphylium leaf blight disease on onion defense-related genes; Table S11: Onion target genes and respective primer sets used for qRT-PCR expression analysis [156].
