# Supplementary material for: Piriformospora indica Primes Onion Response against Stemphylium Leaf Blight Disease
Source: Pathogens. 2021 Aug 26;10(9):1085. doi: 10.3390/pathogens10091085 (PMC8472787; doi:10.3390/pathogens10091085)
Supplement: Supplementary file 1 [file pathogens-10-01085-s001.zip › pathogens-1319137-supplementary .pdf]

pathogens

MDPI

Supplementary Materials

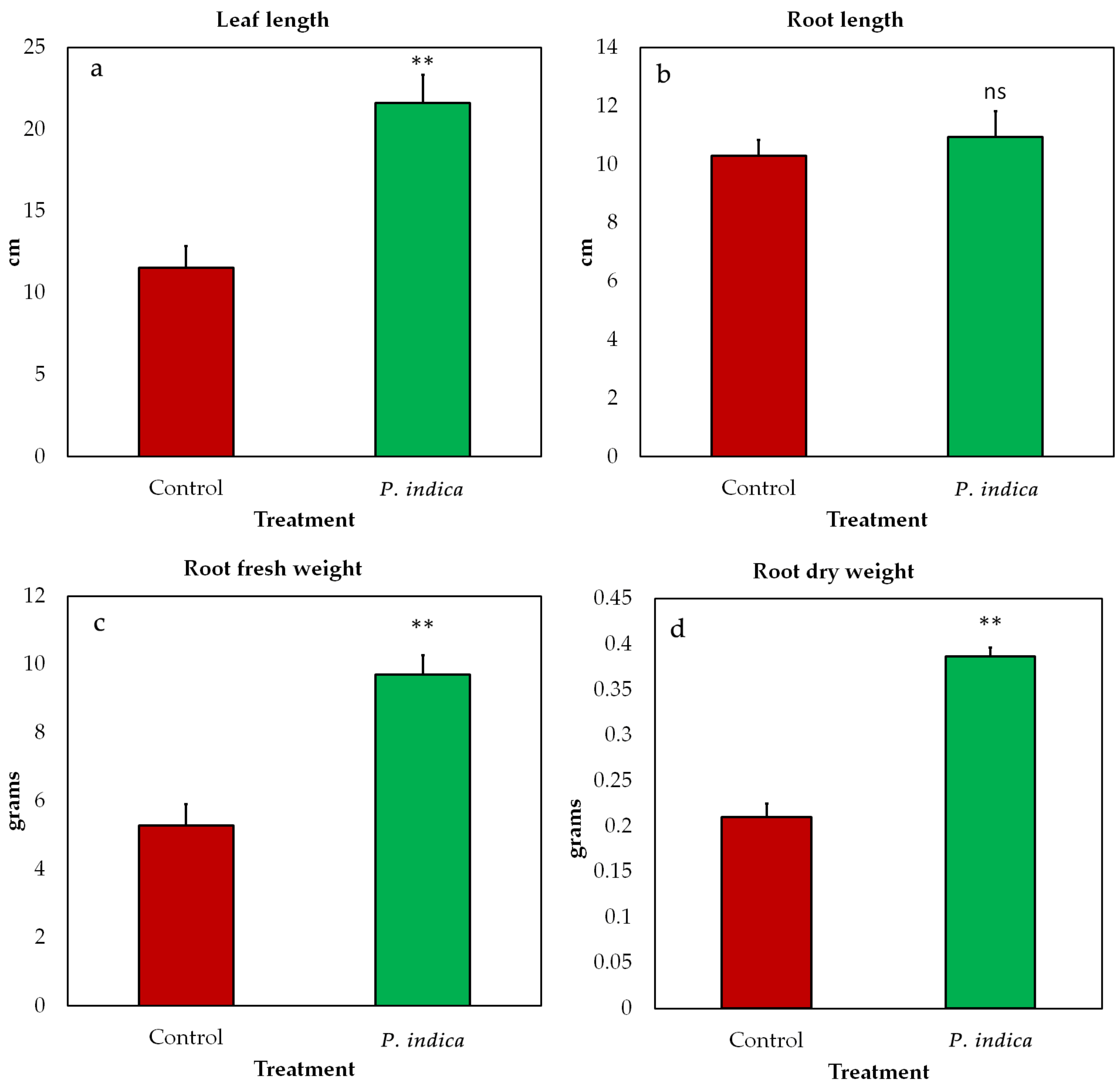

Figure S1. Effects of *P. indica* treatment on onion growth parameters. Leaf length (a), root length (b), fresh (c) and dry (d) weights were measured at 60 days post-treatment and compared with untreated control. Values are expressed as the average of three biological replicates, each consisting of three (a, b) or five plants pooled together (c, d). Statistically significant differences were assessed by Student's t-test at a 5% significance level. *p*-value significance codes: '**' < 0.01, not significant. Error bars indicate standard error of the mean.

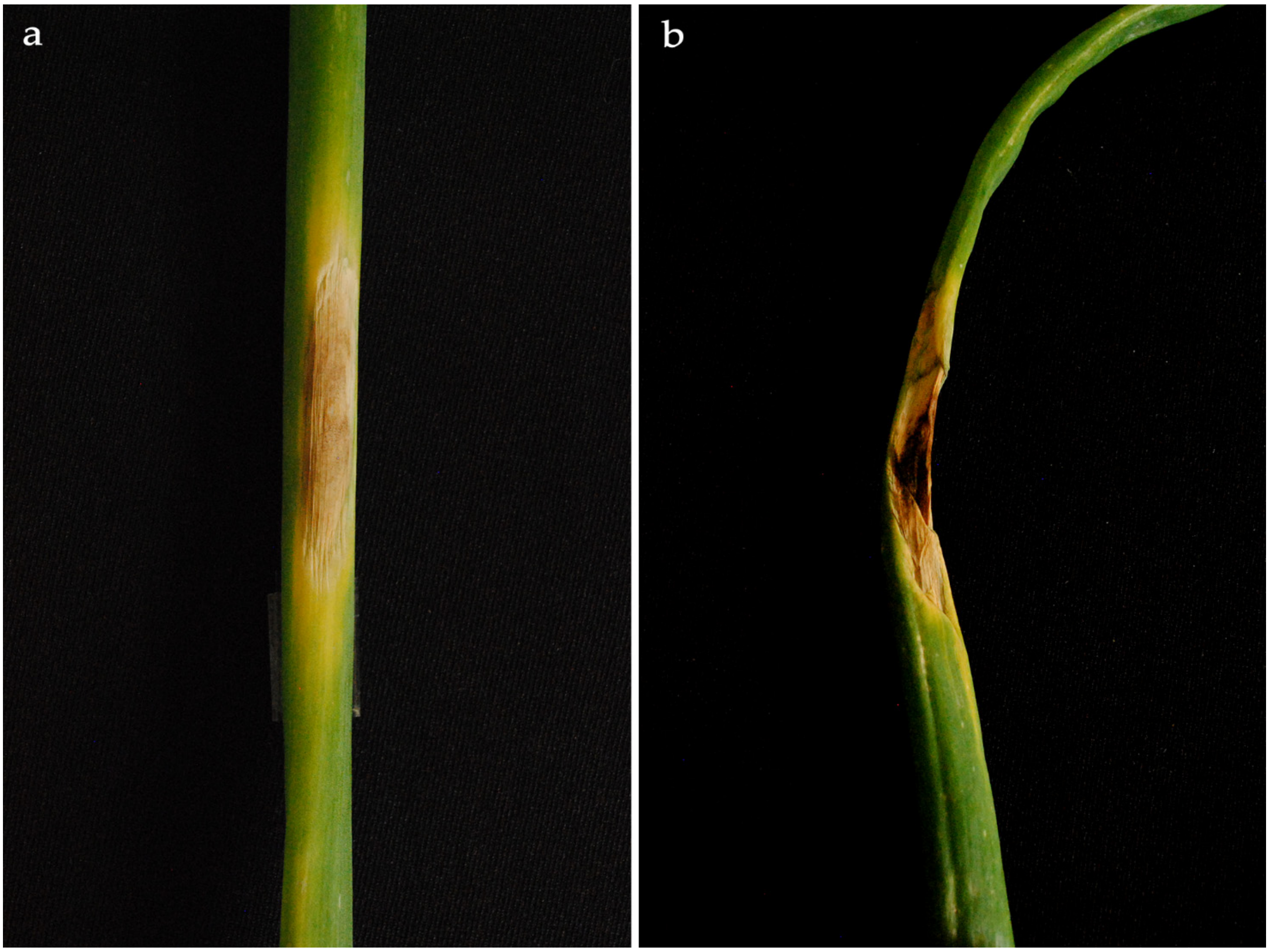

Figure S2. Reduction of Stemphylium leaf blight disease symptoms on onion treated with *P. indica*, compared to the untreated control, as observed 5 days after inoculation with *S. vesicarium*.

Table S1. Results of statistical analysis of the effects of *P. indica* treatment on onion growth parameters.

Treatment	Parameter	*df*	t-value	*p*-value	Mean	Lower CI	Upper CI	Significance
Control<br>*P. indica*	Root length	4.00	−0.60	0.580	10.30<br>10.93	−3.56	2.29	ns
Control<br>*P. indica*	Leaf length	4.00	−4.62	0.010	11.57<br>21.60	−16.07	−4.00	**
Control<br>*P. indica*	Root fresh weight	4.00	−5.18	0.007	5.28<br>9.70	−6.79	−2.05	**
Control<br>*P. indica*	Root dry weight	4.00	−10.07	0.001	0.21<br>0.39	−0.23	−0.13	***

Statistically significant differences were assessed by Student's t-test at a 5% significance level. *p*-value significance codes: '***' < 0.001, '**' < 0.01, 'ns' not significant. *df*: degrees of freedom; CI: confidence interval.

Table S2. Results of statistical analysis of the effects of *P. indica* treatment on Stemphylium leaf blight PDI, in greenhouse conditions.

	dpi	*df*	t-value	*p*-value	Mean	Lower CI	Upper CI	Significance
Untreated/*S. vesicarium*<br>*P. indica*/*S. vesicarium*	1.00	4.00	0.00	1.00E+00	6.67<br>6.67	−8.28	−8.28	ns
Untreated/*S. vesicarium*<br>*P. indica*/*S. vesicarium*	3.00	4.00	4.29	1.28E−02	26.00<br>12.00	4.93	23.07	*
Untreated/*S. vesicarium*<br>*P. indica*/*S. vesicarium*	5.00	4.00	7.34	1.83E−03	40.00<br>18.67	13.27	13.27	**

The percent disease index (PDI) was scored at 1, 3, 5 days post-inoculation with *S. vesicarium* and compared between *P. indica*-treated and untreated plants. Values are expressed as the average of three biological replicates, each consisting of ten plants pooled together. Statistically significant differences were assessed by Student's t-test at a 5% significance level. *p*-value significance codes: '**' < 0.01, '*' < 0.05, 'ns' not significant. dpi: days post-inoculation; *df*: degrees of freedom; CI: confidence interval.

Table S3. Results of ANOVA statistical analysis of the effects of *P. indica* treatment on Stemphylium leaf blight AUDPC, in greenhouse conditions.

	df	t-value	*p*-value	Mean	Lower CI	Upper CI	Significance
Untreated/*S. vesicarium*<br>*P. indica*/*S. vesicarium*	4.00	5.48	0.005	89.67<br>37.33	25.82	78.85	**

The area under disease progress curve (AUDPC) was calculated based on the percent disease index (PDI) scored at 1, 3, 5 days post-inoculation with *S. vesicarium* and compared between *P. indica*-treated and untreated plants. Values are expressed as the average of three biological replicates, each consisting of ten plants pooled together. Statistically significant differences were assessed by Student's t-test at a 5% significance level. *p*-value significance codes: '**' < 0.01, 'ns' not significant. dpi: days post-inoculation; *df*: degrees of freedom; CI: confidence interval.

Table S4. Results of ANOVA statistical analysis of the effects of *P. indica* treatment on Stemphylium leaf blight PDI, in field conditions.

	dat	Year	*df*	Sum of Squares	Mean Squares	F-value	*p*-value	Significance
Treatment	30	2018	4.00	60.27	15.07	1.66	2.34E-01	ns
Residuals			10.00	90.67	9.07			
Treatment	45	2018	4.00	553.60	138.40	13.31	5.15E-04	***
Residuals			10.00	104.00	10.40			
Treatment	60	2018	4.00	1310.90	327.70	30.73	1.36E-05	***
Residuals			10.00	106.70	10.70			
Treatment	75	2018	4.00	1857.60	464.40	39.58	4.22E-06	***
Residuals			10.00	117.30	11.70			
Treatment	30	2019	4.00	252.27	63.07	12.45	6.76E-04	***
Residuals			10.00	50.67	5.07			
Treatment	45	2019	4.00	1140.30	285.07	48.59	1.61E-06	***
Residuals			10.00	58.70	5.87			
Treatment	60	2019	4.00	1906.70	476.70	48.31	1.66E-06	***
Residuals			10.00	98.70	9.90			
Treatment	75	2019	4.00	2875.70	718.90	62.70	4.81E-07	***
Residuals			10.00	114.70	11.50			

Stemphylium leaf blight percent disease index (PDI) was scored at 30, 45, 60, 75 days after field transplanting and compared among plants treated with *P. indica*, treated with three different fungicides or untreated. Values for each year are expressed as the average of three biological replicates, each consisting of ten plants pooled together. Statistically significant differences were assessed by one-way analysis of variance (ANOVA). *p*-value significance codes: '***' < 0.001, 'ns' not significant. dat: days after transplanting; *df*: degrees of freedom.

Table S5. Results of ANOVA statistical analysis of the effects of *P. indica* treatment on Stemphylium leaf blight AUDPC, in field conditions.

	Year	Df	Sum of Squares	Mean Squares	F-value	*p*-value	Significance
Treatment	2018	4.00	3614385.00	903596.00	18.68	1.24E-04	***
Residuals		10.00	483863.00	48386.00			
Treatment	2019	4.00	4480440.00	1120110.00	33.03	9.74E-06	***
Residuals		10.00	339113.00	33911.00			

Stemphylium leaf blight area under disease progress curve (AUDPC) was calculated based on the percent disease index (PDI) scored at 30, 45, 60, 75 days after field transplanting and compared among plants treated with *P. indica*, treated with three different fungicides or untreated. Values for each year are expressed as the average of three biological replicates, each consisting of ten plants pooled together. Statistically significant differences were assessed by one-way analysis of variance (ANOVA). *p*-value significance codes: '***' < 0.001. *df*: degrees of freedom.

Table S6. Results of the linear model analysis of the factors affecting Stemphylium leaf blight PDI, in field conditions.

	df	Sum of Squares	Mean Squares	F-value	*p*-value	Significance
Treatment	4	6487	1621.7	9.23	1.16E-06	***
Year	1	146	146	0.831	0.363	ns
Biological Replicate	1	144	144	0.82	0.367	ns
Residuals	143	25125	175.7			

Stemphylium leaf blight percent disease index (PDI) was scored at 30, 45, 60, 75 days after field transplanting and compared among plants treated with *P.* *indica*, treated with three different fungicides or untreated. The linear model (lm) "PDI ~ Treatment + Year + Biological Replicate" was established to evaluate statistically significant fixed or random effects of the different factors. *p*-value significance codes: '***' < 0.001, 'ns' not significant. df: degrees of freedom.

Table S7. Results of the linear model analysis of the factors affecting Stemphylium leaf blight AUDPC, in field conditions.

	df	Sum of Squares	Mean Squares	F-value	*p*-value	Significance
Treatment	4	7979651	1994913	55.781	3.35E-11	***
Year	1	9720	9720	0.272	0.607	ns
Biological Replicate	2	151361	75681	2.116	0.144	ns
Residuals	22	786787	35763			

Stemphylium leaf blight area under disease progress curve (AUDPC) was calculated based on the percent disease index (PDI) scored at 30, 45, 60, 75 days after field transplanting and compared among plants treated with *P. indica*, treated with three different fungicides or untreated. The linear model (lm) "AUDPC ~ Treatment + Year + Biological Replicate" was established to evaluate statistically significant fixed or random effects of the different factors. *p*-value significance codes: '***' < 0.001, 'ns' not significant. df: degrees of freedom.

Table S8. Results of ANOVA statistical analysis of the effects of *P. indica* treatment and Stemphylium leaf blight disease on onion biochemical response.

	Enzyme	dpi	*df*	Sum of Squares	Mean Squares	F-value	*p*-value	Significance
Treatment	Hydrogen peroxide	1	3	44927.00	14975.70	8.19	8.04E-03	**
Residuals			8	14636.00	1829.40			
Treatment	Hydrogen peroxide	3	3	131410.00	43803.00	149.34	2.32E-07	***
Residuals			8	2347.00	293.00			
Treatment	Hydrogen peroxide	5	3	275123.00	91708.00	352.75	7.78E-09	***
Residuals			8	2080.00	260.00			
Treatment	Lipid peroxidation	1	3	102.71	34.24	6.29	1.69E-02	*
Residuals			8	43.57	5.45			
Treatment	Lipid peroxidation	3	3	591.22	197.07	277.53	2.01E-08	***
Residuals			8	5.68	0.71			
Treatment	Lipid peroxidation	5	3	1323.62	441.21	130.87	3.88E-07	***
Residuals			8	26.97	3.37			
Treatment	Catalase	1	3	35.46	11.82	10.34	3.97E-03	**
Residuals			8	9.14	1.14			
Treatment	Catalase	3	3	32.19	10.73	36.67	5.05E-05	***
Residuals			8	2.34	0.29			
Treatment	Catalase	5	3	26.45	8.82	13.03	1.91E-03	**
Residuals			8	5.41	0.68			
Treatment	Ascorbate peroxidase	1	3	29463.70	9821.20	17.98	6.48E-04	***
Residuals			8	4369.80	546.20			
Treatment	Ascorbate peroxidase	3	3	89546.00	29848.50	47.24	1.96E-05	***
Residuals			8	5055.00	631.80			
Treatment	Ascorbate peroxidase	5	3	144912.00	48304.00	44.95	2.37E-05	***
Residuals			8	8598.00	1075.00			
Treatment	Guaiacol peroxidase	1	3	2291.49	763.83	16.99	7.87E-04	***
Residuals			8	359.61	44.95			
Treatment	Guaiacol peroxidase	3	3	9881.40	3293.80	105.76	8.92E-07	***
Residuals			8	249.20	31.10			
Treatment	Guaiacol peroxidase	5	3	19332.60	6444.20	172.48	1.31E-07	***
Residuals			8	298.90	37.40			
Treatment	Superoxide dismutase	1	3	47665.00	15888.20	29.90	1.07E-04	***
Residuals			8	4252.00	531.50			
Treatment	Superoxide dismutase	3	3	62303.00	20767.70	37.32	4.74E-05	***
Residuals			8	4452.00	556.50			
Treatment	Superoxide dismutase	5	3	71625.00	23875.00	48.18	1.82E-05	***
Residuals			8	3965.00	495.60			
Treatment	Phenylalanine ammonia-lyase	1	3	1.81	0.60	32.61	7.79E-05	***

Residuals			8	0.15	0.02			
Treatment	Phenylalanine ammonia-lyase	3	3	32.16	10.72	25.70	1.85E-04	***
Residuals			8	3.34	0.42			
Treatment	Phenylalanine ammonia-lyase	5	3	60.17	20.06	29.89	1.07E-04	***
Residuals			8	5.37	0.67		8.04E-03	

The biochemical response of selected *A. cepa* enzymes was evaluated at 1, 3, 5 days after *S. vesicarium* or mock inoculation and compared between *P. indica*-treated or untreated plants. Values are expressed as the average of three biological replicates, each consisting of three plants pooled together. Statistically significant differences were assessed by one-way analysis of variance (ANOVA). *p*-value significance codes: '***' < 0.001, '**' < 0.01, '*' < 0.05. dpi: days post-inoculation; *df*: degrees of freedom.

Table S9. Results of the linear model analysis of the factors affecting qRT-PCR expression analysis of onion defense-related genes.

	df	Sum of Squares	Mean Squares	F-value	*p*-value	Significance
Treatment	3	109.8	36.61	4.731	0.00332	**
Experimental Replicate	1	0.10	0.10	0.013	0.90904	ns
Technical Replicate	1	0.0	0.00	0.000	1.00000	ns
Residuals	186	1439.3	7.74			

Relative expression of selected *A. cepa* genes was evaluated at 5 days after *S. vesicarium* or mock inoculation and compared between *P. indica*-treated or untreated plants (factor "Treatment"). The experiment was repeated twice, relying on two independent sets of leaf samples (factor "Experimental replicate"). Each reaction was performed in triplicate wells (factor "Technical replicate"). The linear model (lm) "Relative expression ~ Treatment + Experimental replicate + Technical replicate" was established to evaluate statistically significant fixed or random effects of the different factors. *p*-value significance codes: '**' < 0.01, 'ns' not significant. df: degrees of freedom.

Table S10. Results of ANOVA statistical analysis of the effects of *P. indica* treatment and Stemphylium leaf blight disease on onion defense-related genes.

	Gene	Df	Sum of Squares	Mean Squares	F-value	*p*-value	Significance
Treatment	*AcLOX1*	3	5.90	1.97	8.89	6.30E-03	**
Residuals		8	1.77	0.22			
Treatment	*AcLOX2*	3	18.43	6.14	34.30	6.47E-05	***
Residuals		8	1.43	0.18			
Treatment	*AcWRKY1*	3	6.67	2.22	68.90	4.67E-06	***
Residuals		8	0.26	0.03			
Treatment	*AcWRKY70*	3	6.07	2.02	11.23	3.07E-03	**
Residuals		8	1.44	0.18			
Treatment	*AcPAL1*	3	7.04	2.35	24.77	2.11E-04	***
Residuals		8	0.76	0.09			
Treatment	*AcCHI*	3	28.61	9.54	249.20	3.08E-08	***
Residuals		8	0.31	0.04			
Treatment	*AcGST*	3	13.20	4.40	10.44	3.86E-03	**
Residuals		8	3.37	0.42			

Relative expression of selected *A. cepa* genes was evaluated at 5 days after *S. vesicarium* or mock inoculation and compared between *P. indica*-treated or untreated plants. Each sample was collected from five plants pooled together. Data refer to the most representative of two independent repeated experiments. Each reaction was performed in triplicate wells. Statistically significant differences were assessed by one-way analysis of variance (ANOVA). *p*-value significance codes: '***' < 0.001, '**' < 0.01, . dpi: days post-inoculation; df: degrees of freedom.

Table S11. Onion target genes and respective primer sets used for qRT-PCR expression analysis.

Target gene	GenBank Accession		Primer Sequence	Amplicon Size (bp)
AcLOX1	KU363822.1	Fwd	5′-AGGCACGGCAGTGTTAATGA -3′	204
		Rev	5′-CACCAGCCGCTACAGATGAT-3′	
AcLOX2	KX427168.1	Fwd	5′-CGAGCAACCGATGGCAAAAT-3′	187
		Rev	5′-TTCCGGTGATGAACTGCTCC-3′	
AcCHI	KM114296.1	Fwd	5′-CCTACGCCCAACAAAGTG-3′	192
		Rev	5′-ATACTCCTGATCCTCCTCC-3′	
AcGST	AB300334.1	Fwd	5′-TCGTGAGAGTGATTGCGGTT-3′	210
		Rev	5′-CGCAGGTAGGAGGTCTGTTC-3′	
AcPAL1	KF421110.1	Fwd	5′-AGGTGGAAGTTGTAAGGGCG-3′	187
		Rev	5′-CCATTGCAAACCGCCTCAAA-3′	
AcWRKY1	KY273103.1	Fwd	5′-CCTGTGCCATTGAGACCTTT-3′	208
		Rev	5′-GTGCATGTGCTGAATTGCTT-3′	
AcWRKY70	*	Fwd	5′-TGGAGAGTTCGCTGGTCAAA-3′	210
		Rev	5′-TACAGCCTCTGCGAGAAACG-3′	
AcACT	GU570135.2	Fwd	5′-GCACCAAGAGCAGTATTC-3′	183
		Rev	5′-CCAAATCTTCTCCATGTCA-3′	

* Primers for amplification of *AcWRKY70* gene were designed based on the onion transcriptome dataset made available by Ghodke et al. [156].
